# IQ Motif-Containing GTPase-Activating Protein 2 (IQGAP2) Is a Novel Regulator of Colonic Inflammation in Mice

**DOI:** 10.1371/journal.pone.0129314

**Published:** 2015-06-05

**Authors:** Amr M. Ghaleb, Agnieszka B. Bialkowska, Ashley J. Snider, Dmitri V. Gnatenko, Yusuf A. Hannun, Vincent W. Yang, Valentina A. Schmidt

**Affiliations:** 1 Department of Medicine, Stony Brook University, Stony Brook, New York, United States of America; 2 Northport Veterans Affairs Medical Center, Northport, New York, United States of America; 3 Stony Brook Cancer Center, Stony Brook University, Stony Brook, New York, United States of America; 4 Genomics Core Facility, Stony Brook University, Stony Brook, New York, United States of America; French National Centre for Scientific Research, FRANCE

## Abstract

IQ motif-containing GTPase-activating protein 2 (IQGAP2) is a multidomain scaffolding protein that plays a role in cytoskeleton regulation by juxtaposing Rho GTPase and Ca^2+^/calmodulin signals. While IQGAP2 suppresses tumorigenesis in liver, its role in pathophysiology of the gastrointestinal tract remains unexplored. Here we report that IQGAP2 is required for the inflammatory response in colon. Mice lacking *Iqgap2* gene (*Iqgap2^-/-^* mice) were resistant to chemically-induced colitis. Unlike wild-type controls, *Iqgap2^-/-^* mice treated with 3% dextran sulfate sodium (DSS) in water for 13 days displayed no injury to colonic epithelium. Mechanistically, resistance to colitis was associated with suppression of colonic NF-κB signaling and IL-6 synthesis, along with diminished neutrophil and macrophage production and recruitment in *Iqgap2^-/-^* mice. Finally, alterations in IQGAP2 expression were found in colons of patients with inflammatory bowel disease (IBD). Our findings indicate that IQGAP2 promotes inflammatory response at two distinct levels; locally, in colonic epithelium through TLR4/NF-κB signaling pathway, and systemically, via control of maturation and recruitment of myeloid immune cells. This work identifies a novel mechanism of colonic inflammation mediated by signal transducing scaffolding protein IQGAP2. IQGAP2 domain-specific blocking agents may represent a conceptually novel strategy for therapy of IBD and other inflammation-associated disorders, including cancer.

## Introduction

The goal of this study was to determine the contribution of IQ motif-containing GTPase-activating protein 2 (IQGAP2) to inflammation of the gastrointestinal tract, the central disease-causing feature of inflammatory bowel disease (IBD). IBD has been estimated to affect up to 1.4 million people in the United States [1], although its incidence is underreported due to the fact that the onset of illness is often gradual and the differential diagnosis is broad. The causes and mechanisms of this debilitating disorder are not well understood, and curative agents are not available. Both genetic and environmental factors influence susceptibility to IBD. This applies to both major forms of IBD, ulcerative colitis (UC) and Crohn’s disease (CD).

IQGAP2 is a 180 kDa cytoplasmic multidomain scaffolding protein that belongs to a protein family consisting of three highly homologous members, IQGAP1, IQGAP2 and IQGAP3 [2]. The domain structure of IQGAPs includes an actin-binding calponin homology (CH) domain, a WW domain, four IQ motifs, and a GTPase-binding domain (GBD) responsible for binding Rho GTPases Rac1 and cdc42 [2]. Despite their similar structure, IQGAP1 and IQGAP2 appear to play opposing roles *in vivo*. IQGAP1 is the most extensively studied member of the family and has been identified as a *bona fide* oncogene that promotes tumorigenesis in various cancers, including colorectal [3–5]. In contrast, IQGAP2 acts as a tumor suppressor in the liver by regulating Wnt/β-catenin and PI3K/Akt signaling [6, 7]. Mice lacking both *Iqgap1* and *Iqgap2* display relative protection against hepatocellular carcinoma (HCC) and longer survival compared to *Iqgap2* ^*-/-*^ mice, implying that at least in the liver IQGAP1 antagonizes activity of IQGAP2 [6]. The tumor suppressing repertoire of IQGAP2 has been recently expanded to two additional cancer types: gastric [8, 9] and prostate [10]. IQGAP2 has also been shown to be important in platelets as an integrator of Rho GTPase and Ca^2+^/calmodulin signals with cell adhesive and cytoskeletal reorganizational events [11]. Yet, physiological functions of IQGAP2 remain understudied and its role in chronic inflammation and pathogenesis of IBD has not been addressed.

Here we show for the first time that ablation of the *Iqgap2* gene leads to protection from dextran sulfate sodium (DSS)-induced colitis in mice. Lack of inflammatory response observed in *Iqgap2* ^*-/-*^ colon appears to be multifactorial and may be explained by the inhibited colonic Toll-like receptor 4 (TLR4)/NF-κB signaling and subsequent abrogation of IL-6 synthesis, along with diminished neutrophil and macrophage production and their recruitment to the site of injury.

This study thus describes a novel role of IQGAP2 in mediating the inflammatory and immune response of the colon and provides new insights into the mechanisms of IBD pathogenesis.

## Materials and Methods

### Ethics statement

The use of murine models and experimental protocols within this study was approved by the Stony Brook University Institutional Animal Care and Use Committee. The study involving de-identified patient colon biopsy specimens reported here has been reviewed and approved by the Institutional Review Board at Stony Brook University before the study began. The IRB waived the need for written informed consent from the participants per 45CFR46.116.d.

### Mice

Generation of *Iqgap2* ^*-/-*^ conventional knockout mouse model was described previously [6]. Briefly, in order to generate the *Iqgap2* null allele, a 36,241-bp genomic fragment of the mouse *Iqgap2* gene spanning exons 18 to 30 and corresponding to the IQ3 and IQ4 motifs and GBD domain was replaced with a neomycin resistance gene via site-specific homologous recombination. *Iqgap2* ^*-/-*^ progeny were born at normal Mendelian ratios, were fertile and clinically normal. *Iqgap2* ^*-/-*^ mice were maintained on 129 genetic background. Female 8- to 16 week old wild-type (WT) and *Iqgap2* ^*-/-*^ mice were used in all animal experiments. Mice were maintained on a regular 12-hour light-dark cycle under standard conditions and were provided with food and water *ad libitum*.

### Patient colonic biopsy specimens

Paired human colitis and normal colon needle biopsy specimens obtained from seven patients archived the Stony Brook University BioBank (http://www.stonybrookmedical/center.org/pathology/biobank) were kindly provided by Dr. Jennie Williams (Stony Brook University). The study involving de-identified patient colon biopsy specimens reported here has been reviewed and approved by the Institutional Review Board at Stony Brook University before the study began. The samples were de-identified prior to release to the researchers and qualified for a waiver of consent per 45CFR46.116.d. In this cohort, two patients were diagnosed with ulcerative colitis and five with Crohn’s disease. The average subjects’ age was 36 years and the M/F ratio was 2/5.

### DSS treatment and colitis clinical scoring

For acute colitis induction, age-matched WT and *Iqgap2* ^*-/-*^ mice were given 3% (wt/vol) DSS (MP Biomedicals, Solon, OH) in water *ad libitum* for up to 13 days. Control mice received regular water without DSS. Mice were divided into two groups: one group was euthanized on day 13 of DSS treatment, and the other group was allowed to recover from DSS treatment by replacing DSS on day 13 with regular water for 7 more days. Mice in both groups were weighed daily and changes in body weight were calculated as follows: body weight change (%) = [(weight on a given day (days 0–13)—weight on day 0)/weight on day 0] X 100. Mice also were monitored daily for stool consistency and signs of occult bleeding. Upon euthanasia, the entire large intestines were removed and the distance from the ileocecal junction to the rectum was measured as a marker of the severity of colitis. Colitis clinical scoring, expressed as a disease activity index (DAI), utilized a system that combines weight loss, stool consistency and signs of intestinal bleeding [12]. Each category’s score ranged from 0 to 4, resulting in a minimal colitis clinical score of 0 and a maximal score of 12.

### Histology, immunohistochemistry and immunofluorescence

The entire colon from the cecum to the rectum of WT and *Iqgap2* ^*-/-*^ mice was removed, flushed with modified Bouin’s fixative (50% ethanol, 5% acetic acid), and cut-open longitudinally for gross examination. The colons were then Swiss-rolled and incubated in 10% formalin overnight at room temperature for subsequent embedding in paraffin wax, hematoxylin and eosin (H&E) staining and histological analysis. Images of colon sections were captured using a Nikon Eclipse *90i* microscope. To identify goblet cells, the Alcian Blue Staining Kit (Biocare Medical) was used.

For immunohistochemistry (IHC), 5-μm colonic tissue sections were de-paraffinized in xylene, incubated in 3% hydrogen peroxide in methanol at room temperature for 30 minutes, rehydrated in ethanol gradient (100%, 95%, 70%), and treated with 10 mM Na citrate buffer, pH 6.0, at 120°C for 10 minutes in a pressure cooker. Sections were then incubated in a blocking buffer (5% BSA in TBS-Tween) at 37°C for 1 hour, followed by treatment with primary antibodies at 4°C overnight in a humidified chamber. The following primary antibodies were used: mouse monoclonal IQGAP1 (BD Biosciences) at 1:300 dilution; rabbit monoclonal IQGAP2 (D1X8, Cell Signaling) at 1:200; rabbit polyclonal NF-κB (p65) (sc-372, Santa Cruz Biotechnologies) at 1:300; mouse monoclonal phospho-STAT3 (Tyr705) (Cell Signaling) at 1:200; rabbit polyclonal Chromogranin A (CgA) (Epitomics) at 1:500; goat polyclonal carbonic anhydrase-1 (CA-1) (Santa Cruz) at 1:500; mouse monoclonal TLR4 (abcam) at 1:200; and rabbit polyclonal MyD88 (sc-11356, Santa Cruz) at 1:300. Next day, sections were washed and incubated with HRP-conjugated secondary antibodies for 30 min at 37°C. Betazoid DAB (Biocare Medical, CA, USA) was used to reveal IHC staining in tissues. Slides were counterstained with hematoxylin, dehydrated and mounted in Cytoseal-XYL mounting medium (Fisher Scientific).

For immunofluorescence (IF), tissue sections were processed as above prior to incubation with primary antibodies. The antibodies included untagged rabbit polyclonal antibodies against CC3 (Cell Signaling) and Ki67 (Novocastra, Leica Microsystems, IL), both at 1:200, and also PE-conjugated rat anti-mouse IL-6 (BD Biosciences) at 1:100, rat anti-mouse AlexaFluor 488-conjugated F4/80 (Invitrogen) at 1:100, FITC-conjugated rat anti-mouse Ly-6G (BD Biosciences) at 1:100, and FITC-conjugated hamster anti-mouse CD11c (BD Biosciences) at 1:100. For double IF staining, two primary antibodies, specific for IL-6 and either F4/80 or Ly-6G, were used simultaneously at 1:100 dilution each. To detect untagged CC3 and Ki67 primary antibodies, anti-rabbit AlexaFlour 488-conjugated secondary antibody (Invitrogen) was used. To visualize nuclei, slides were counterstained with DAPI (Fisher Scientific). Slides were mounted in Prolong gold antifade (Life Technologies) and examined under a Nikon Eclipse *90i* microscope, and representative images were captured.

For quantification of IL-6, F4/80, Ly-6G, CD11c, CC3, Ki67 and CgA expression in colonic mucosa, positive cells were counted in at least 6 randomly selected fields at 200 X by two independent investigators who were blinded to mouse genotype and treatment.

### RNA extraction and real-time quantitative RT-PCR

Total RNA was isolated from fresh colonic tissue using the TRIzol method (Invitrogen Life Technologies). Alternatively, total RNA from formalin-fixed paraffin embedded (FFPE) colons was extracted using FFPE RNeasy kit (Qiagen) according to the manufacturer’s instructions. Quantitative RT-PCR was performed using 10 ng of RNA, QuantiTect SYBR Green RT-PCR Kit and QuantiTect Primer Assays, cat. #QT00098875 for mouse IL-6, and cat. #QT00106169 for mouse IL-10 (Qiagen), as per manufacturer’s protocol. The mRNA abundance was determined from triplicate assays performed in parallel for each primer pair and calculated using the comparative threshold cycle number [13]. The abundance of mRNA of the target genes was normalized to β-actin expression. Relative expression of genes of interest was measured using the ΔΔCt method [14].

### Protein extraction and immunoblotting

Freshly isolated colons were flushed with phosphate-buffered saline (PBS), cut longitudinally and scraped to isolate colonic mucosa. Tissues were homogenized and sonicated in lysis buffer (50 mM Tris, 150 mM NaCl, 1% NP40, 0.5% Na deoxycholate) supplemented with protease inhibitors. After centrifugation at 35,000 g for 15 min, 10 μg of the supernatants were separated on 4–15% gradient SDS-polyacrylamide gels (Bio-Rad) and transferred onto nitrocellulose membranes (Bio-Rad) and probed at 4°C overnight with the following mouse monoclonal primary antibodies: IQGAP1 (BD Biosciences) at 1:1000 dilution; IQGAP2 (sc-55525, Santa Cruz) at 1:1000; β-actin and α-tubulin (Sigma Aldrich) at 1:2000. Blots were washed and probed with anti-mouse HRP-conjugated secondary antibodies at 37°C for 1 hour. Chemiluminescent detection was used to visualize protein bands.

### Statistical analysis

For all analyses, differences in means between groups were analyzed by a two-tailed Student’s *t* test using a *P* value of 0.05 as a measure of statistical significance. Error bars in graphs denote SEM unless otherwise indicated. Meta-analysis of IQGAP2 expression in various human and mouse RNA microarray datasets was conducted using the following online tools: GEO2R (www.ncbi.nlb.nih.gov), Illumina NextBio data analysis platform (www.nextbio.com), and ExpressionBlast platform (www.expression.cs.cmu.edu/index.html).

## Results

### *Iqgap2* ^*-/-*^ colons display goblet cell hyperplasia

**Fig 1:**
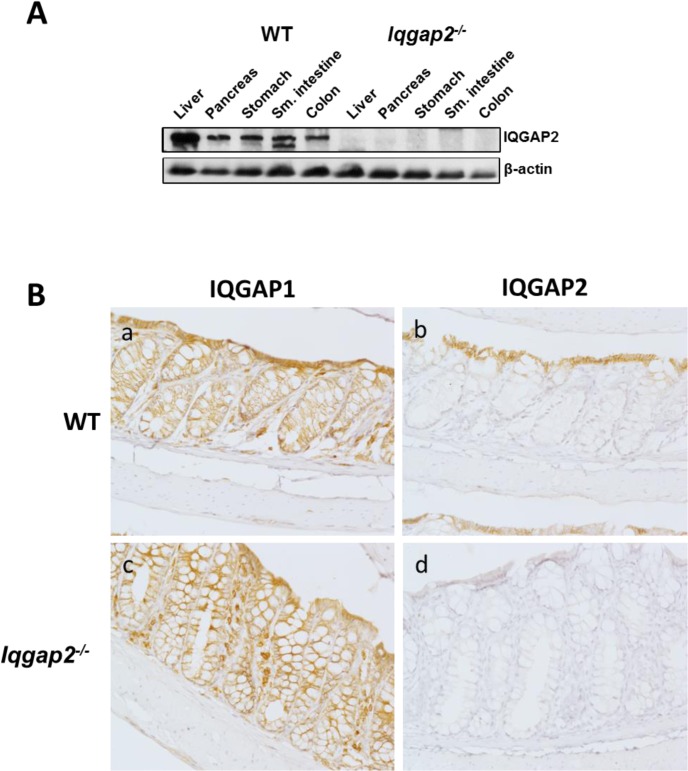
IQGAP2 expression in organs of the digestive system. **A.** Immunoblot of digestive tract organs from wild-type (WT) and *Iqgap2* ^*-/-*^ mice, probed for IQGAP2 and β-actin as a control for equal protein loading. Representative blots of N = 3 per genotype are shown. **B.** IHC of IQGAP1 and IQGAP2 in WT (panels a, b) and *Iqgap2* ^*-/-*^ (panels c, d) mouse colon. Magnification is 200X.

Despite their high degree of homology, IQGAP1 and IQGAP2 proteins display distinct expression levels in WT mouse digestive tract (Fig 1A and S1 Fig). Both isoforms are expressed in the colon and, noteworthy, *Iqgap2* ^*-/-*^ colons do not show a compensatory increase in the levels of IQGAP1 (S1 Fig). At the cellular level, IQGAP1 appeared to be ubiquitous in the colonic epithelium, showing positive staining at membrane and cytoplasm, while IQGAP2 expression was restricted to the lateral and basolateral sides of the luminal terminally differentiated colonic epithelial cells (Fig 1B). IQGAP2 seems to be dispensable for normal colonic epithelial homeostasis based on unaltered baseline morphology of *Iqgap2* ^*-/-*^ colons. The exception is mucin-secreting goblet cells in *Iqgap2* ^*-/-*^ colonic crypts (S2 Fig). The number of goblet cells per crypt was found to be elevated up to 2-fold in *Iqgap2* ^*-/-*^ colons when compared to WT controls. Individual goblet cells also appeared enlarged in *Iqgap2* ^*-/-*^ colonic crypts (S2 Fig). This implies that mucin biogenesis and/or goblet cell differentiation may be abnormal in *Iqgap2* ^*-/-*^ colons and IQGAP2 may be required for tight control of intestinal epithelial differentiation, which requires further investigation.

### Complete loss of *Iqgap2* gene in mice leads to protection from DSS-induced colitis

IQGAP2 has been shown to have an anti-proliferative effect *in vitro* in human prostate cancer [10] and hepatocellular carcinoma [15] cell lines. It was hypothesized here that ablation of IQGAP2 may induce cell proliferation, and the effect may be distinct in colon due to its significant levels of IQGAP2 expression. Increased proliferation rates, in turn, may be beneficial for the repair of colonic injury and defense against colitis. *Iqgap2* ^*-/-*^ knockout mouse model represented the unique tool to test this hypothesis.

**Fig 2:**
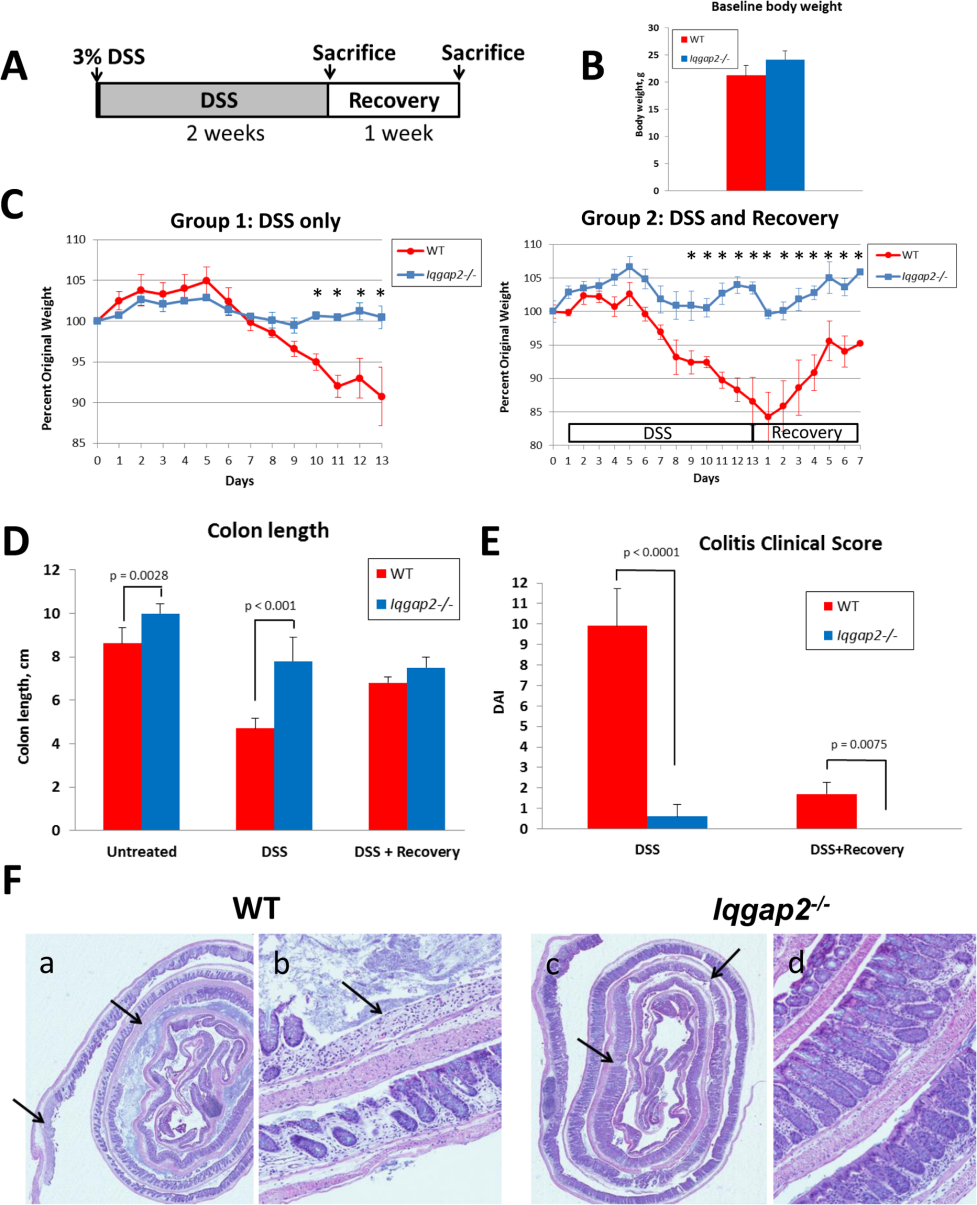
Physiological and histological evidence of resistance to DSS-induced colitis in *Iqgap2* ^*-/-*^ mice. **A.** Schema of a DSS treatment experiment. DSS at the concentration of 3% was administered in drinking water for up to 13 days. A separate group of mice was treated with 3% DSS for 13 days and then allowed to recover on regular water for 7 days. **B.** Baseline body weight of untreated WT and *Iqgap2* ^*-/-*^ mice. The difference between genotypes is not statistically significant (p = 0.1059). **C.** In contrast to the WT control group, *Iqgap2* ^*-/-*^ mice did not lose weight as a result of DSS treatment (left panel); after a 7-day recovery period, WT mice restored 95% of their baseline weight before DSS treatment (right panel). Data are presented as the mean ± SEM. Statistically significant (p < 0.05) differences between genotypes are indicated with asterisks. Resistance of *Iqgap2* ^*-/-*^ mice to experimental colitis is also demonstrated by colon length (**D**) and low colitis disease activity index (DAI) (**E**). **F.** Histological evidence (H&E) of the absence of colitis in *Iqgap2* ^*-/-*^ mice. While DSS treatment resulted in an expansive colonic epithelium loss (black arrows) in WT colons (panels a, b), *Iqgap2* ^*-/-*^ colons were minimally affected (panels c, d). N = 5 per group per experiment; experiment was repeated three times; the results of one representative experiment are shown.

To elucidate a physiological role of IQGAP2 in the colon, *Iqgap2* ^*-/-*^ mice, along with WT controls, were subjected to 3% DSS in drinking water for up to 13 days to induce acute colitis as outlined in Fig 2A. DSS-induced colitis is a well-established animal model of mucosal inflammation that is widely used in preclinical studies of IBD [16]. The extended duration of DSS treatment was chosen because mice of the 129 genetic background are known to be less sensitive to DSS compared to C57BL/6 strain, which usually develops severe colitis within 7 days of treatment [17]. The extent of DSS-induced colitis was measured by assessment of colon length and clinical scoring. Of note, *Iqgap2* ^*-/-*^ mice in these experiments had slightly higher baseline body weight compared to WT, although the difference was not statistically significant (Fig 2B). At day 8 of DSS treatment, WT mice began showing signs of diarrhea and weight loss (Fig 2C, left panel), while *Iqgap2* ^*-/-*^ mice maintained their regular weight and normal stool consistency. At day 10, fecal occult blood was detected in WT, but not in *Iqgap2* ^*-/-*^ mice. By day 13, the termination point of DSS experiment, WT mice lost 10% of total body weight (Fig 2C, left panel) and 46% of the colon length on average (Fig 2D), while the body weight of *Iqgap2* ^*-/-*^ mice remained unchanged compared to the baseline levels before treatment. *Iqgap2* ^*-/-*^ mice displayed a mild (16%) decrease in the colon length upon DSS treatment. While the baseline colon length of the untreated *Iqgap2* ^*-/-*^ mice was found to be slightly higher than that of WT controls (Fig 2D), which is probably attributable to the somewhat larger size of *Iqgap2* ^*-/-*^ mice, normalization to the baseline (untreated) levels nevertheless confirms that DSS treatment causes significantly more severe loss of colon length in WT compared to *Iqgap2* ^*-/-*^ mice (S3 Fig). Overall colitis clinical score was determined at 10 ± 1.8 for WT mice (with 12 being the maximal possible score) and 0.6 ± 0.7 for *Iqgap2* ^*-/-*^ mice, indicating severe colitis in WT and its absence in *Iqgap2* ^*-/-*^ mice (Fig 2E). At the histological level, DSS caused extensive tissue damage in WT colons, characterized by extensive areas of colonic epithelium loss and ulceration (Fig 2F, panels a, b). *Iqgap2* ^*-/-*^ colonic epithelium remained essentially undamaged (Fig 2F, panels c, d). Remarkably, in the colon of WT mice exposure to DSS up-regulated IQGAP2 protein levels and also altered its distribution, expanding its expression pattern from the luminal colonic epithelial cells in untreated controls, to colonocytes throughout the entire length of the crypt in DSS-treated WT mice (S4 Fig). This may reflect a potential involvement of IQGAP2 in a cellular response to the oxidative stress triggered by DSS treatment. Overall, *Iqgap2* ^*-/-*^ mice displayed significant resistance to DSS-induced colitis as evident by maintenance of a normal body weight, intact colonic morphology and lack of clinical symptoms.

To determine whether IQGAP2 also plays a role during recovery from DSS-induced colonic injury, separate groups of *Iqgap2* ^*-/-*^ and WT mice were allowed to recover for 7 days after DSS treatment. Consistent with the results in the acute colitis model described above, *Iqgap2* ^*-/-*^ mice showed no signs of colitis after a 7-day recovery period, while WT mice showed only a partial recovery, with body weights at 95% of the basal levels and small unrepaired areas of colonic epithelium still present (Fig 2C, right panel and S5 Fig).

### *Iqgap2* ^*-/-*^ colons display suppressed NF-κB signaling

**Fig 3:**
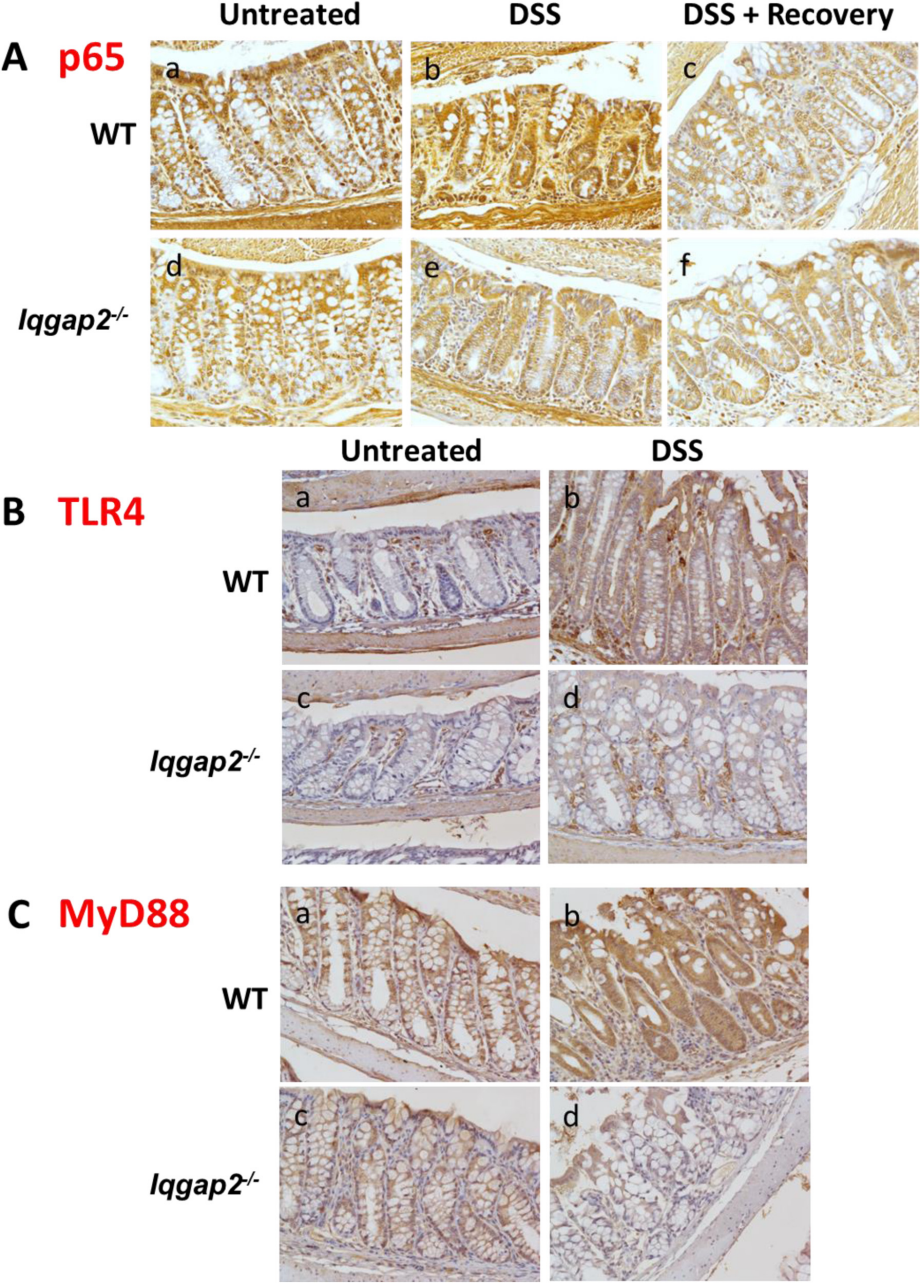
Suppression of NF-κB signaling in *Iqgap2* ^*-/-*^ colons. **A.** IHC shows decreased baseline levels of p65 subunit of NF-κB in *Iqgap2* ^*-/-*^ colon compared to WT (panels a, d). While DSS treatment resulted in elevated levels of p65 in WT colon, it failed to elicit the same response in *Iqgap2* ^*-/-*^ colon (panel b, e). Termination of DSS treatment results in a restoration of the baseline p65 levels within 7 days in both genotypes (panel c, f). **B.** IHC of TLR4 in WT and *Iqgap2* ^*-/-*^ colons before and after DSS treatment. **C.** IHC of MyD88 in the same samples. Note low levels of both TLR4 and MyD88 in *Iqgap2* ^*-/-*^ colons after DSS exposure (panels d). A representative image of N = 5 per genotype is shown for each IHC. Magnification is 200 X.

Activation of NF-κB signaling has been detected in colonic biopsy samples from patients with both UC and CD [18] and also in mouse colons affected by DSS-induced colitis [19]. Additionally, it has been shown that IQGAP2 physically interacts with NF- κB *in vitro* [20]. Since *Iqgap2* ^*-/-*^ mice demonstrate a strong protective phenotype against DSS insult, activity of NF-κB pathway was assessed next in this mouse model. Immunohistochemistry (IHC) revealed that expression levels of the p65 subunit of NF-κB were diminished in *Iqgap2* ^*-/-*^ colon compared to WT at baseline in untreated animals (Fig 3A, panels a, d). DSS had no effect on p65 levels in *Iqgap2* ^*-/-*^ colons, while it markedly up-regulated p65 expression in colons of WT mice, a finding consistent with a DSS-induced inflammatory response reported in mice (Fig 3A, panels b, e). A seven-day recovery period following DSS exposure restored colonic p65 levels to baseline levels seen before DSS treatment in WT colons, whereas its levels in *Iqgap2* ^*-/-*^ colons were unchanged (Fig 3A, panels c, f).

NF-κB stability and activation are primarily regulated by signaling through TLRs [21], a class of pattern recognition receptors involved in the adaptive and innate immune responses [22]. TLR4 is expressed in the colon and its aberrant signaling has been implicated in IBD [23]. It is the main receptor for gram-negative bacterial lipopolysaccharide (LPS) in the mouse gut [24]. It is also a major TLR in macrophages [25]. To further investigate the observed suppression of NF-κB activation in *Iqgap2* ^*-/-*^ colons, levels of TLR4 and its adaptor protein MyD88 were assessed by IHC. The baseline colonic levels of both TLR4 and MyD88 were indistinguishable between genotypes in untreated animals. Interestingly, while DSS caused overexpression of TLR4 and MyD88 in WT colons, the levels of these proteins remained low in *Iqgap2* ^*-/-*^ colons (Fig 3B and 3C, respectively).

### IL-6 production in response to DSS is suppressed in *Iqgap2* ^*-/-*^ colon

**Fig 4:**
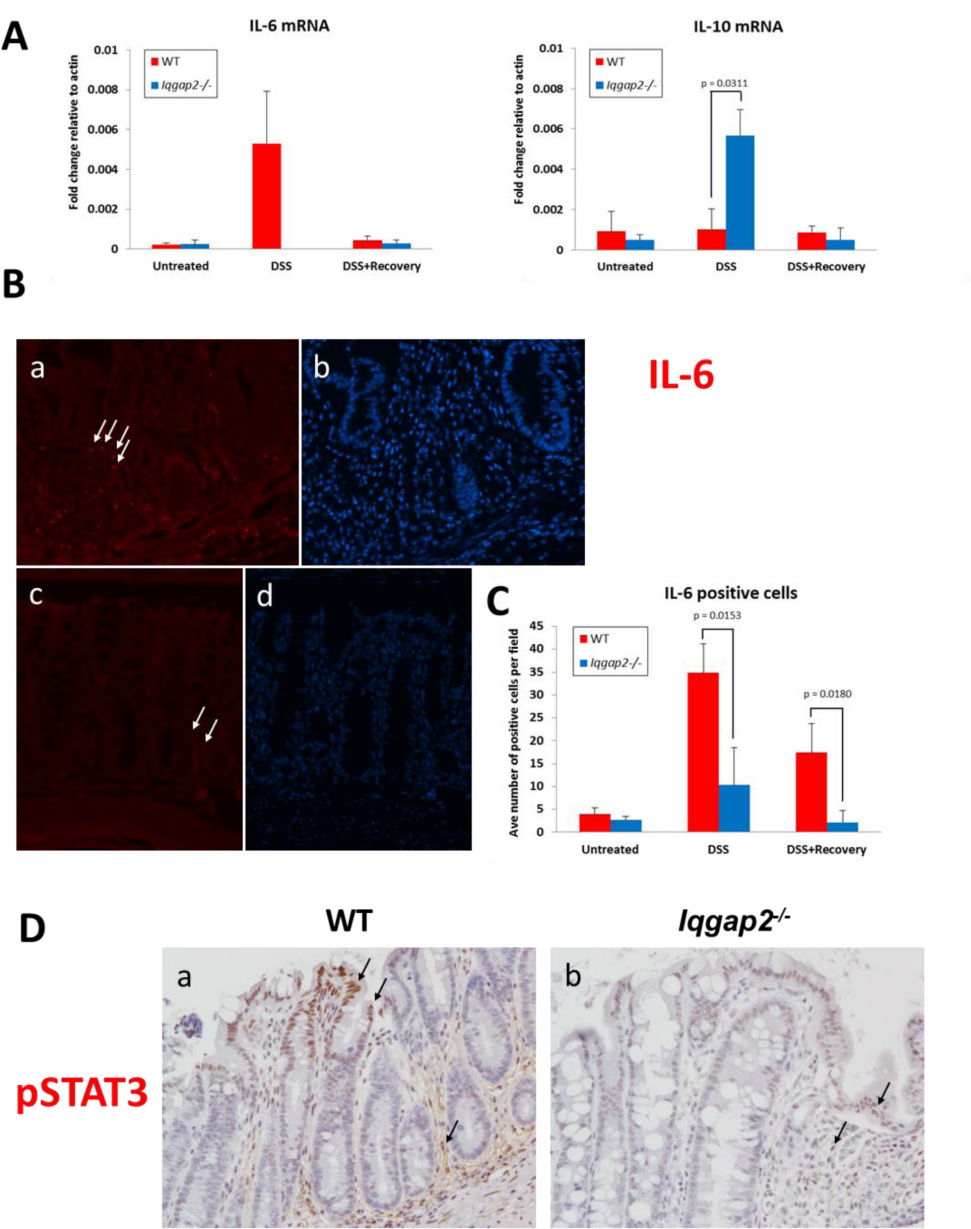
*Iqgap2* ^*-/-*^ colons are characterized by diminished production of IL-6 in response to DSS treatment. **A.** IL-6 (left) and IL-10 (right) mRNA cytokine levels as quantified by qRT-PCR in colons from WT and *Iqgap2* ^*-/-*^ mice from the three groups: untreated, treated with DSS, and treated with DSS and allowed to recover for 7 days, N = 3 per group per genotype. The levels of IL-6 mRNA in DSS-treated *Iqgap2* ^*-/-*^ colons were beyond the sensitivity of the method used. Data are presented as a transcript fold change relative to actin mRNA transcript levels. **B.** IF showing reduced IL-6 production (red) in DSS-treated *Iqgap2* ^*-/-*^ colons (panel c) compared to DSS-treated WT (panel a). White arrows indicate representative IL-6-positive cells. Panels b and d show corresponding DAPI staining. Magnification is 200 X. Images are representative of N = 3 per genotype. **C.** Quantification of IF IL-6 positive cells in colons from WT and *Iqgap2* ^*-/-*^ mice from the three groups: untreated, treated with DSS, and treated with DSS and allowed to recover for 7 days. Data represent an average of ten randomly selected fields per sample ± SD. N = 3 per genotype/treatment. P-values indicating statistically significant differences are shown. **D.** IHC for phospho-STAT3(Tyr705) in WT and *Iqgap2* ^*-/-*^ colons before and after DSS treatment, N = 5 per group. Representative pSTAT3-positive cells are pointed with black arrows. Magnification is 200 X.

NF-κB realizes its central role in the inflammatory response in part through the control of *Il-6* gene expression [26]. One of the proposed mechanisms of IBD involves inappropriate response to resident microbes in the gastrointestinal tract that leads to overproduction of many pro-inflammatory cytokines, including IL-6 [27]. Exposure to DSS has been shown to affect levels and activity of numerous cytokines in colonic mucosa and immune cells in mice [28]. We therefore proposed that the observed *Iqgap2* ^*-/-*^ phenotype may be a result of an inhibited pro-inflammatory cytokine production in these mice. To test this, cytokine expression profile was analyzed by qRT-PCR in untreated, DSS-treated and DSS+Recovery colonic tissue samples from *Iqgap2* ^*-/-*^ and WT mice. Before DSS treatment, there were no differences in colonic mRNA levels of IL-6 and IL-10, important modulators of colitis-induced inflammatory response [29], between the genotypes (Fig 4A). However, while DSS induced expression of colonic IL-6 over 20 fold in WT mice compared to untreated WT controls, it failed to stimulate IL-6 production in colons of *Iqgap2* ^*-/-*^ mice (Fig 4A, left graph). Conversely, mRNA levels of anti-inflammatory cytokine IL-10 were 5-fold higher in *Iqgap2* ^*-/-*^ colons compared to WT in response to DSS treatment (Fig 4A, right graph). Both IL-6 and IL-10 expression returned to the baseline levels in WT and *Iqgap2* ^*-/-*^ colons following a seven-day recovery period after DSS treatment (Fig 4A). Next, IHC in colon sections using an IL-6-specific antibody showed a significant increase in the numbers of IL-6-positive cells in DSS-treated WT compared to DSS-treated *Iqgap2* ^*-/-*^ mice (Fig 4B and 4C).

In the colon, IL-6 realizes its pro-proliferative and anti-apoptotic effects through its key mediator, transcription factor STAT3 [30, 31]. IL-6 binding to its receptors causes activation of STAT3 via phosphorylation by JAK2 kinase [32]. Consistent with decreased IL-6 production, upon DSS treatment *Iqgap2* ^*-/-*^ colons had a very low number of cells positive for STAT3 phosphorylated at Tyr705 compared to DSS-treated WT controls (Fig 4D), which is indicative of lack of STAT3 activation. WT colons displayed intense nuclear pSTAT3 staining in areas of epithelial damage from DSS-induced colitis and, unlike *Iqgap2* ^*-/-*^ colons, in the stroma. Interestingly, upon DSS treatment, *Iqgap2* ^*-/-*^ colonic mucosa demonstrated levels of apoptosis similar to those of WT controls as evident by numbers of cleaved caspase-3 (CC3)-positive cells (S6A and S6B Fig). However, significant reduction of apoptosis levels compared to WT controls was detected in *Iqgap2* ^*-/-*^ colons after a seven-day recovery period following DSS exposure (S6 Fig). There were also no significant differences in epithelial cell proliferation levels between the genotypes based on Ki67 IHC before or after DSS treatment (S7 Fig). Normalization to the levels in untreated colons revealed no difference in the number of Ki67-positive cells between genotypes in the DSS+Recovery group (S7 Fig).

### *Iqgap2* ^*-/-*^ mice have decreased levels of macrophages and neutrophils in comparison to WT mice

**Fig 5:**
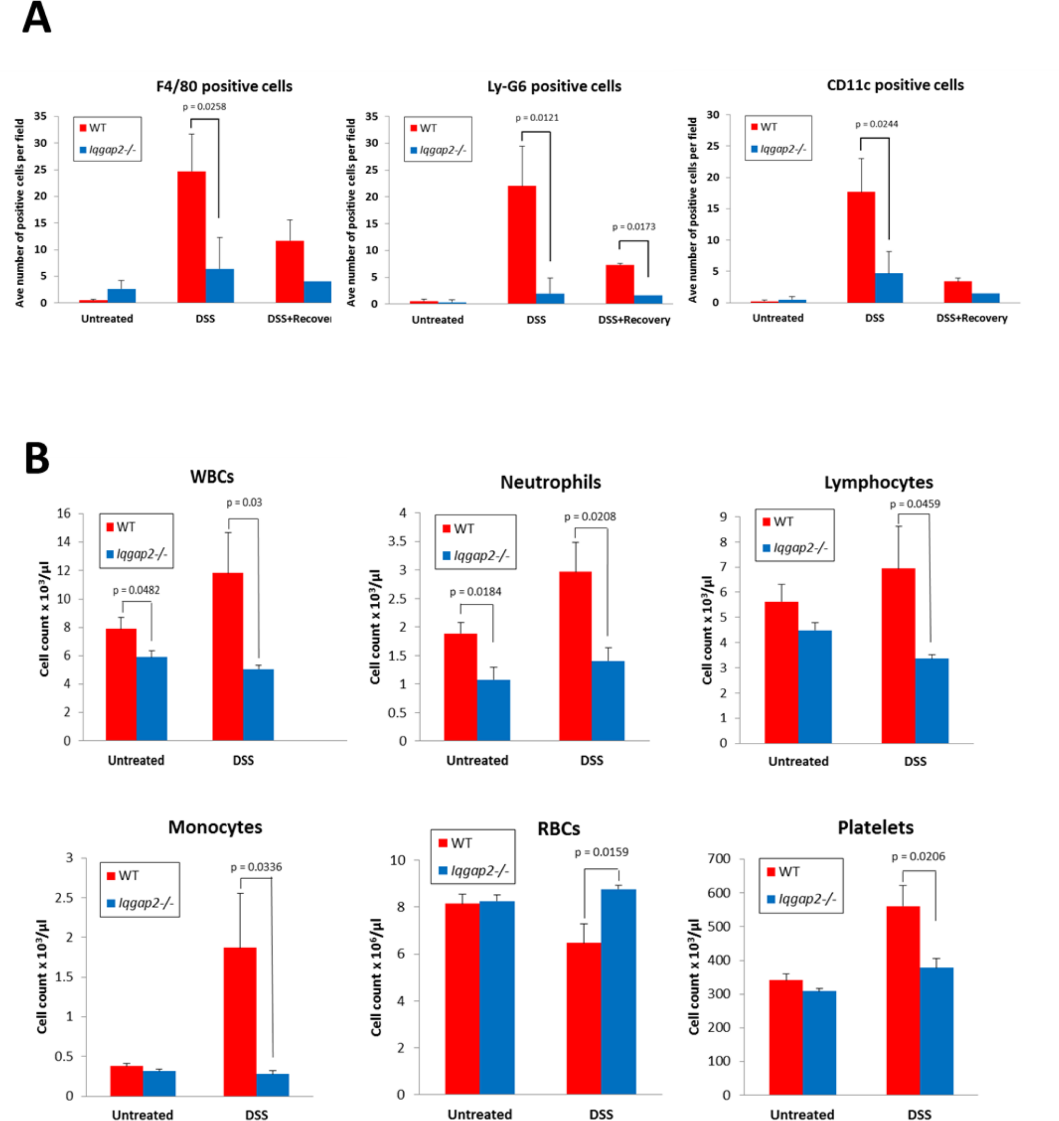
Reduced production of white blood cells (WBC) in *Iqgap2* ^*-/-*^ mice. **A.** Quantification of positive cells as a result of IF shows significantly decreased numbers of infiltrating macrophages (F4/80), neutrophils (Ly-6G) and dendritic cells (CD11c) in *Iqgap2* ^*-/-*^ colons compared to WT following DSS treatment. The number of positive fluorescent cells was obtained by counting cells in six randomly selected fields per colon sample from WT and *Iqgap2* ^*-/-*^ mice from the three groups: untreated, treated with DSS, and treated with DSS and allowed to recover for 7 days. **B.** Complete blood count (CBC) confirms reduced numbers of neutrophils, lymphocytes and monocytes in circulation in *Iqgap2* ^*-/-*^ mice treated with DSS. Data are presented as the mean ± SEM. N = 3 per genotype/treatment (**A**), N = 5 per genotype/treatment (**B**), p-values indicating statistically significant differences are shown.

In intestine, the major IL-6 producers are dendritic cells, macrophages and T-cells [30]. The contribution of innate immunity to IBD remains an area of intense debate. Macrophages and dendritic cells are considered important factors in regulating the onset of IBD. DSS-induced colitis in mice also has been shown to be driven primarily by myeloid innate immune cells, as disease can occur in T and B cell-deficient mice [33]. To determine whether diminished IL-6 levels in *Iqgap2* ^*-/-*^ colonic mucosa upon exposure to DSS reflect either lower numbers of infiltrating immune cells or inhibited activity of these cells (hence inability to produce IL-6), IHC was conducted in *Iqgap2* ^*-/-*^ and WT colon sections from untreated, DSS-treated and DSS+Recovery groups of mice using markers specific to macrophages (F4/80), neutrophils (Ly-6G) and dendritic cells (CD11c) (Fig 5A and S8 and S9 Figs). The numbers of cells positive for the three markers studied were indistinguishable between WT and *Iqgap2* ^*-/-*^ colons from untreated mice (Fig 5A). However, colonic mucosa from WT mice showed a significant (up to 10-fold) increase in infiltration by all three myeloid cell types following DSS treatment (Fig 5A), yet *Iqgap2* ^*-/-*^ colons were largely devoid of these infiltrating immune cells. The nature of IL-6-producing cells in colons studied was confirmed by double IF staining simultaneously using antibodies against IL-6 and either F4/80 or Ly-6G (S9 Fig). As a result, both colonic macrophages and neutrophils were identified as IL-6-producing in mice of both genotypes in these experiments. These findings suggest a defect in either immune cell recruitment/homing or their maturation in *Iqgap2* ^*-/-*^ mice.

Analysis of complete blood counts (CBCs) revealed that DSS-treated *Iqgap2* ^*-/-*^ mice had significantly lower numbers of circulating white blood cells (WBC), including neutrophils, monocytes and lymphocytes, compared to DSS-treated WT controls (Fig 5B and S10 Fig). The baseline numbers of WBC and neutrophils were also significantly lower in untreated *Iqgap2* ^*-/-*^ mice compared to untreated WT mice (Fig 5B). Lastly, the number of macrophages isolated from bone marrow of untreated *Iqgap2* ^*-/-*^ mice was 3-fold lower compared to untreated WT controls (data not shown). This suggests the possibility of an earlier unexplored defect in immune cell maturation in *Iqgap2* ^*-/-*^ mice, which also could explain their protection against DSS-induced colitis. Importantly, bone marrow-derived *Iqgap2* ^*-/-*^ macrophages showed functional competency comparable to WT controls, as indicated by similar levels of secreted IL-6 and IL-10 and migration ability (not shown). Collectively, these results point to an essential role of IQGAP2 in promoting colonic inflammation and, on a larger scale, in hematopoiesis and innate immunity.

### IQGAP2 expression is altered in human colitis

**Fig 6:**
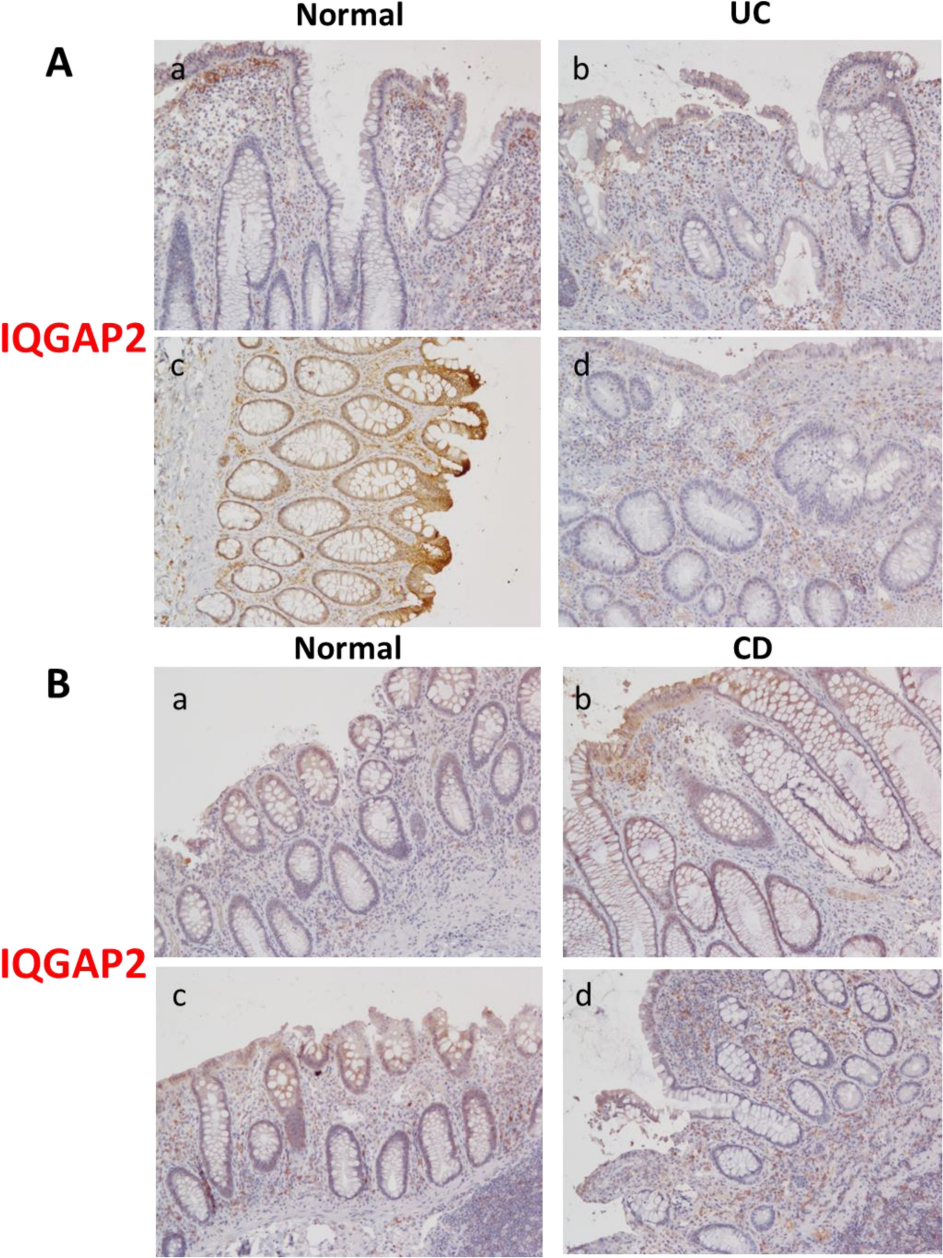
IQGAP2 levels in colon specimens of patients with IBD. **A.** Two cases of ulcerative colitis (UC): panels a and b represent Case #1, and panels c and d–Case #2. **B.** Two cases of Crohn’s disease (CD), panels are designated as above. Images are representative of the total of 7 IBD patient cases. Magnification is 200 X.

To our knowledge, this is the first study addressing the role of IQGAP2 in colonic inflammation and there are no data available on how IBD affects the IQGAP2 protein. To establish relevance of the *Iqgap2* ^*-/-*^ colitis model to human disease, IQGAP2 protein levels were compared between colonic tissue affected by IBD (both UC and CD) and matching normal colonic tissue from a small cohort of seven patients. IHC revealed that IQGAP2 expression is distinct between the two forms of IBD. IQGAP2 levels were reduced in UC specimens compared to normal mucosa (Fig 6A), yet they were moderately elevated in CD specimens versus normal tissue (Fig 6B). Importantly, infiltrating immune cells, macrophages and neutrophils in particular, were among the cells with the most increased levels of IQGAP2 in inflamed CD colonic mucosa (Fig 6B). Even in UC colons, IQGAP2 diminished expression seemed to be limited to colonic epithelium in comparison to matching unaffected tissue, while IQGAP2 levels were unchanged or elevated in myeloid cell infiltrates (Fig 6A). This hints the possibility that IQGAP2 involvement in colonic inflammation is compartmentalized, with its major role realized through control of myeloid immune cells rather than through colonic epithelium.

To gain further insight into IQGAP2 expression in human IBD and colorectal cancer (CRC), we conducted an extensive meta-analysis of published RNA microarray datasets. The results of this analysis are summarized in S1 Table. Among eight different studies, which had data on *Iqgap2* available, comparing RNA transcript expression profiles of IBD colonic biopsies and either healthy controls or adjacent unaffected colonic tissue, *Iqgap2* levels were minimally decreased in IBD versus either healthy or “unaffected” colonic tissue. While the fold-change decrease was small, ranging from -1.21 to -1.8, the change was consistently statistically significant (S1 Table). UC and CD samples were indistinguishable in terms of *Iqgap2* RNA expression. One study out of four focusing on human CRC, which had data on *Iqgap2* available, showed a moderate increase (+2.64 fold change, p = 0.0036) in *Iqgap2* RNA transcript expression in CRC liver metastasis compared to primary CRC tumors (S1 Table).

## Discussion

IBD is a multifactorial disease and the molecular mechanisms driving its pathogenesis are still not fully understood. Previous studies identified IQGAP2 as a tumor suppressor in liver and stomach and as a mediator of several major signaling pathways [reviewed in [34]], although until now its role in gastrointestinal inflammation has not been addressed. Here we report that IQGAP2 is required for the development of acute colitis in mice. We found that *Iqgap2* ^*-/-*^ mice were protected from colonic injury in the DSS-induced colitis model. Protection from colitis in *Iqgap2* ^*-/-*^ mice was evident by maintenance of normal body weight, absence of hematochezia and intact colonic epithelium and crypt architecture. While IQGAP2 appears dispensable for normal colonic homeostasis, upon exposure to DSS, *Iqgap2* ^*-/-*^ colonic mucosa displayed suppressed NF-κB activation and low levels of TLR4, MyD88, IL-6 and pSTAT3(Tyr705) compared to mucosa from DSS-treated WT mice. Yet, *Iqgap2* ^*-/-*^ colonic cell proliferation and apoptosis rates were similar to those of WT controls. Moreover, our results also indicate that *Iqgap2* ^*-/-*^ mice had significantly less myeloid infiltrating cells in colons and lower number of circulating white blood cells, including neutrophils and monocytes. The only morphological aberration observed in *Iqgap2* ^*-/-*^ colons was hyperplasia of goblet cells irrespective of DSS treatment.

The *Iqgap2*-deficient mouse studied here is a whole body knockout model, which allowed us to uncover IQGAP2 complex function in both colon-specific and systemic inflammatory response. Still, dissecting the precise molecular mechanisms of IQGAP2 involvement in inflammation will require generation of tissue-specific *Iqgap2*-deficient models. Based on the results of this study, and also the ability of the IQGAP2 scaffold to play the role of a signal transducer in multiple signaling pathways and the recent report of IQGAP2’s ability to directly bind NF-κB [20], we propose that IQGAP2 modulates inflammatory response by functioning as an adaptor protein in the TLR4/NF-κB signaling pathway in both colonic epithelial and stromal cells. We hypothesize that IQGAP2 may positively regulate NF-κB stability and activation through spatial and/or temporal control of MyD88, Rac1 or Akt. NF-κB activation has been shown to occur in a Rac1/PI3K-dependent manner [35–37]. IQGAP2 may regulate NF-κB signaling by stabilizing Rho GTPase Rac1. A recent study in a large cohort of IBD patients identified a single nucleotide polymorphism (SNP) rs10951982 in the Rac1 gene leading to increased Rac1 expression and higher susceptibility to IBD [38]. The same study also showed that a conditional deletion of Rac1 in mouse neutrophils and macrophages resulted in these mice being protected from DSS-induced colitis. Therefore IQGAP2 interaction with Rac1 may be crucial for its role in colonic inflammation.

It is also feasible to propose that IQGAP2 realizes its pro-inflammatory action through interaction with p38 (MAPK) or ERK1/2, both involved in control of NF-κB and cytokine production. While the list of the confirmed binding partners of IQGAP2 remains relatively short, its extensively studied homolog IQGAP1 binds ERK1/2 through its WW domain and MEK1/2 and Akt through the IQ motifs [39, 40]. Since IQGAP1 and IQGAP2 IQ motifs share 72% of their amino acids, it is conceivable that IQGAP2 is capable of binding these kinases as well. Finally, recent studies have implicated the Hippo signaling pathway in self-renewal and repair of the intestinal epithelium [41, 42]. We reported recently that *Iqgap2* ^*-/-*^ livers display strong activation of Yes-associated protein (YAP), a downstream transcriptional co-activator of the Hippo pathway [43], although given the normal levels of cell proliferation in *Iqgap2* ^*-/-*^ colons, IQGAP2 involvement in colonic Hippo signaling seems unlikely.

Expression of TLRs by intestinal epithelial cells is generally low, but during intestinal inflammation it increases in all forms of IBD [44]. Consistently, in the current study, TLR4 and MyD88 levels were elevated in WT colons in response to DSS treatment in our experiments, but not in *Iqgap2* ^*-/-*^ colons. This may be explained by documented lack of inflammation in *Iqgap2* ^*-/-*^ colons, as well as by a positive feedback loop driven by cytokines whose levels are also low. On the other hand, IQGAP2-mediated regulation of TLR4 expression is also possible. It also would be of interest to assess the contribution of gut microbiota in *Iqgap2* deficiency to NF-κB expression levels and further studies with germ-free *Iqgap2* ^*-/-*^ mice are warranted to address this.

An imperative question that arose from our study is whether IQGAP2’s function in inflammation is compartmentalized in the body, with it playing distinct roles in colonic epithelium versus immune cells. Our results show that IQGAP2 may be capable of both: aiding the development of colitis through a direct function in colonic epithelium and in a more indirect manner, through modifying neutrophil/macrophage maturation, their inflammatory recruitment and migration, and overall innate immune response. CBCs of *Iqgap2* ^*-/-*^ mice (Fig 5B), IQGAP2 protein expression pattern observed in human colitis biopsy specimens (Fig 6) and data on the conditional deletion of Rac1 in mouse neutrophils and macrophages [38] suggest that the neutrophil/macrophage axis may be the predominant one in IQGAP2’s pro-inflammatory function. Tissue-specific phenotypes of macrophages are the result of irreversible differentiation programs that are controlled by lineage-specific transcription regulators or, as an alternative, they can be based on reversible functional polarization programs controlled by multiple transcriptional regulators [45]. IQGAP2 may be such a regulator, temporally and spatially controlling macrophage maturation and polarization programs in response to specific stimuli in different tissues. IQGAP2 may realize this function through the TLR4/NF-κB pathway, since TLR interactions are known to trigger macrophage maturation [46]. Significant hyperplasia of goblet cells in *Iqgap2* ^*-/-*^ colons may also be attributed to control of their differentiation by IQGAP2. Of note, we observed that in *Iqgap2* ^*-/-*^ colons the baseline levels of CgA and CA-1, an endocrine cell marker and a differentiation marker of colonic enterocytes, respectively, were the same as in WT controls, although upon exposure to DSS, *Iqgap2* ^*-/-*^ colons had ~ 2.5-fold more cells positive for CgA, (S11 Fig), suggesting an up-regulated endocrine cell differentiation in response to DSS. It remains to be further investigated whether goblet cell hyperplasia alone could be responsible for the protective phenotype of *Iqgap2* ^*-/-*^ mice. Bone marrow transplants or conditional tissue-specific mice will be employed in the future to distinguish the contribution of hematopoietic vs. extra-hematopoietic cells to the IQGAP2-mediated inflammatory response.

Finally, we speculated that IQGAP2 expression levels in colon may be linked to susceptibility to IBD, and may also be distinct in UC vs. CD. The human *Iqgap2* gene is located at chromosome 5, region q13. Thus far, studies into disease-associated mutations in the IQGAP2 gene are lacking. Our pilot analysis of IQGAP2 protein expression in a small cohort of IBD patient colon biopsy specimens produced mixed results (Fig 6).We were able to determine reduced levels of IQGAP2 protein in colonic mucosa of UC specimens (N = 2) compared to normal mucosa. This trait was not evident in CD specimens (N = 5). The mechanisms regulating *Iqgap2* gene expression remain largely unexplored, although there is emerging evidence that epigenetic factors may be involved. A microarray gene expression profile of an intestine-specific conditional knockout mouse with deleted histone deacetylase 2 (*Hdac2*) gene encoding a transcriptional repressor revealed a modest increase in *Iqgap2* expression compared to WT controls [47] (S1 Table). Similarly, miR-21-deficient mice showed a small increase in *Iqgap2* expression in colonic mucosa versus WT controls [48], suggesting the possibility of regulation of *Iqgap2* expression by miRNAs. Lastly, intriguing data have emerged from *MyD88* ^*-/-*^ mouse studies. It was shown that azoxymethane (AOM)-DSS-induced colitis triggered up-regulation of *Iqgap2* expression in *MyD88* ^*-/-*^ colons and not in the similarly treated WT controls [49] (S1 Table), suggesting that MyD88 or its downstream effectors may negatively regulate IQGAP2 expression.

Unchanged or even modestly decreased levels of *Iqgap2* RNA transcript observed in human colitis specimens can be interpreted as follows. Our IHC results in IBD colonic specimens (Fig 6) show that significant changes in IQGAP2 protein expression may be occurring in infiltrating myeloid cells rather than in epithelium and the microarray studies reviewed here do not distinguish between cell types. Further studies of IQGAP2 protein expression in larger specimen cohorts of human IBD of different types and stages are needed to determine whether IQGAP2 plays a role in initiation or maintenance of colonic inflammation, as well as to address IQGAP2 protein stability and epigenetic mechanisms possibly regulating IQGAP2 expression.

In summary, the current study identifies IQGAP2 as a novel regulator of colonic inflammation and also provides evidence that it may play a role in the systemic immune response through control of macrophage maturation and recruitment to the site of injury. Signaling scaffolding proteins such as IQGAP2 represent amenable therapeutic targets due to their domain structure. Synthetic IQGAP2 domain-specific inhibiting peptides make attractive candidates for investigation of potential immunomodulatory properties, since their action would constitute a selective blockage of IQGAP2 interactions with specific binding partners rather than ablation of the entire functional spectrum of IQGAP2. Of note, the first successful inhibition of IQGAP1 homolog using WW domain-mimicking synthetic peptides has been recently reported [50]. The peptides effectively blocked IQGAP1 interaction with ERK1/2, resulting in inhibition of tumorigenesis in mouse models for melanoma, breast and pancreatic cancers. Another study has demonstrated the utility of IQGAP2 IQ motif-mimicking peptides as anti-bacterial agents [51].

In addition to IBD, our findings may be applicable to other chronic inflammation-associated disorders, including diabetes, cardiovascular diseases, osteoporosis, and cancer.

## Supporting Information

S1 Fig IQGAP1 protein expression in organs of mouse digestive tract. **A.** Immunoblot showing IQGAP1 expression in organs of mouse digestive tract. **B.** Densitometric quantification of IQGAP1 protein levels in WT vs. *Iqgap2* ^*-/-*^ organs normalized to α-tubulin.
(TIF)

S2 Fig *Iqgap2* ^*-/-*^ colons display a higher number and size of goblet cells compared to WT controls. **A.** H&E (panels a, b) and Alcian Blue (panels c, d) stainings. Representative images of N = 3 per genotype are shown. Characteristic crypts with distinct size of goblet cells are circled. **B.** Numbers of goblet cells were counted per crypt in six random fields and data presented as mean ± SD. A p-value indicating significant difference is shown.
(TIF)

S3 Fig Normalized colon length. Colon length of WT and *Iqgap2* ^*-/-*^ mice from two treatment groups, DSS-treated and DSS+Recovery, was normalized to colon length of untreated mice of respective genotypes.
(TIF)

S4 Fig DSS exposure causes up-regulation of IQGAP2 protein expression in WT mice. Representative images of N = 5 are shown. Magnification is 200 X.
(TIF)

S5 Fig H&E staining of colon sections from WT and *Iqgap2* ^*-/-*^ mice from the DSS+Recovery group. N = 5 per genotype; experiment was repeated three times; the results of one representative experiment are shown. Panels b and d show higher magnification of samples in panels a and c. An unrepaired area of epithelium loss is marked with a black arrow.
(TIF)

S6 Fig Apoptosis analysis of mouse colons after DSS treatment. **A.** DSS induces apoptosis at a similar rate in *Iqgap2* ^*-/-*^ colons compared to WT controls as evident by IF using cleaved caspase-3 (CC3) antibody. Representative images of colon sections from untreated and DSS-treated WT (panels a, c) and *Iqgap2* ^*-/-*^ (panels b, d) mice are shown. Representative images of colons from the DSS+Recovery group (panels e, f) are also shown. Images are overlays of CC3 IF (green) and DAPI (blue) staining. Images are representative of N = 3 per group. White arrows indicate characteristic CC3-positive cells. **B.** Quantification of CC3-positive cells in WT and *Iqgap2* ^*-/-*^ colons from mice from the three treatment groups: untreated, DSS-treated and DSS and Recovery. CC3-positive cells were counted in crypts in six random fields. Data are presented as mean ± SD per crypt. A p-value indicating statistically significant difference is shown. **C.** Numbers of CC3-positive cells in colons from the DSS and DSS+Recovery groups normalized to numbers of CC3-positive cells in untreated colons.
(TIF)

S7 Fig Cell proliferation analysis of mouse colons after DSS treatment. **A.** Ki67 IF of colons from the untreated, DSS-treated and DSS+Recovery mouse groups. Images are overlays of Ki67 IF (green) and DAPI (blue) staining. Images are representative of N = 3 per group. **B.** Quantification of Ki67-positive cells was conducted as described for CC3 in S6 Fig. Magnification is 200 X. **C.** Numbers of Ki67-positive cells in colons from the DSS-treated and DSS+Recovery groups normalized to numbers of Ki67-positive cells in untreated colons.
(TIF)

S8 Fig Immunofluorescent staining of mouse colons for F4/80, Ly-6G and CD11c. Representative images of IF for F4/80 (panels a, b), Ly-6G (panels c, d) and CD11c (panels e, f) using colon sections from DSS-treated WT and *Iqgap2* ^*-/-*^ mice, N = 3 per group. Images are overlays of IF with antibodies of interest (green) and DAPI (blue) staining. Magnification is 200 X.
(TIF)

S9 Fig Double Immunofluorescent staining of mouse colons for IL-6 and either F4/80 (A) or Ly-6G (B). Representative images of IF for IL-6 (red, panels a, e), either F4/80 or Ly-6G (green, panels b, f), and DAPI (blue, panels c, g) using colon sections from DSS-treated WT and *Iqgap2* ^*-/-*^ mice are shown, N = 3 per group. Images in panels d, h are overlays of three stainings: IL-6, either F4/80 or Ly-6G, and DAPI. White arrows indicate characteristic positive cells. Magnification is 200 X.
(TIF)

S10 Fig Normalized CBCs of WT and *Iqgap2* ^*-/-*^ mice. CBCs of WT and *Iqgap2* ^*-/-*^ mice from the DSS-treated group were normalized to CBCs of untreated mice of respective genotypes.
(TIF)

S11 Fig Immunohistochemical staining of mouse colons for Chromogranin A (CgA) and carbonic anhydrase-1 (CA-1). **A.** Representative images of WT and *Iqgap2* ^*-/-*^ colons untreated and after DSS treatment, N = 5 per group per genotype. Magnification is 200 X. Characteristic positive cells are pointed with black arrows. **B.** Quantification of CgA-positive cells. Positive cells were counted in six random fields. Data are presented as mean ± SD. P-values indicating statistically significant difference are shown. **C.** IHC of carbonic anhydrase-1 (CA-1) in the same samples as in **A**.
(TIF)

S1 Table Meta-analysis of various RNA microarray datasets. Meta-analysis was conducted to assess changes in *Iqgap2* mRNA levels in human IBD, CRC, and mouse DSS- and AOM-DSS-induced colitis models.
(DOCX)

## References

[1] Loftus EV Jr. Clinical epidemiology of inflammatory bowel disease: Incidence, prevalence, and environmental influences. Gastroenterology. 2004;126(6):1504–17. . 15168363 10.1053/j.gastro.2004.01.063

[2] White CD, Brown MD, Sacks DB. IQGAPs in cancer: a family of scaffold proteins underlying tumorigenesis. FEBS Lett. 2009;583(12):1817–24. Epub 2009/05/13. doi: S0014-5793(09)00373-1 [pii] 10.1016/j.febslet.2009.05.007 19433088 PMC2743239

[3] Nabeshima K, Shimao Y, Inoue T, Koono M. Immunohistochemical analysis of IQGAP1 expression in human colorectal carcinomas: its overexpression in carcinomas and association with invasion fronts. Cancer Lett. 2002;176(1):101–9. . 11790459 10.1016/s0304-3835(01)00742-x

[4] Briggs MW, Li Z, Sacks DB. IQGAP1-mediated stimulation of transcriptional co-activation by beta-catenin is modulated by calmodulin. J Biol Chem. 2002;277(9):7453–65. . 11734550 10.1074/jbc.M104315200

[5] Tang MC, Chan LC, Yeh YC, Chen CY, Chou TY, Wang WS, et al Thymosin beta 4 induces colon cancer cell migration and clinical metastasis via enhancing ILK/IQGAP1/Rac1 signal transduction pathway. Cancer Lett. 2011;308(2):162–71. 10.1016/j.canlet.2011.05.001 . 21621326

[6] Schmidt VA, Chiariello CS, Capilla E, Miller F, Bahou WF. Development of hepatocellular carcinoma in Iqgap2-deficient mice is IQGAP1 dependent. Mol Cell Biol. 2008;28(5):1489–502. 10.1128/MCB.01090-07 18180285 PMC2258764

[7] Gnatenko DV, Xu X, Zhu W, Schmidt VA. Transcript profiling identifies iqgap2(-/-) mouse as a model for advanced human hepatocellular carcinoma. PLoS One. 2013;8(8):e71826 Epub 2013/08/21. 10.1371/journal.pone.0071826 23951254 PMC3741273

[8] Jin SH, Akiyama Y, Fukamachi H, Yanagihara K, Akashi T, Yuasa Y. IQGAP2 inactivation through aberrant promoter methylation and promotion of invasion in gastric cancer cells. Int J Cancer. 2008;122(5):1040–6. . 17957782 10.1002/ijc.23181

[9] Fan B, Dachrut S, Coral H, Yuen ST, Chu KM, Law S, et al Integration of DNA copy number alterations and transcriptional expression analysis in human gastric cancer. PLoS One. 2012;7(4):e29824 Epub 2012/04/28. 10.1371/journal.pone.0029824 22539939 PMC3335165

[10] Xie Y, Yan J, Cutz JC, Rybak AP, He L, Wei F, et al IQGAP2, A candidate tumour suppressor of prostate tumorigenesis. Biochim Biophys Acta. 2012;1822(6):875–84. Epub 2012/03/13. 10.1016/j.bbadis.2012.02.019 . 22406297

[11] Schmidt VA, Scudder L, Devoe CE, Bernards A, Cupit LD, Bahou WF. IQGAP2 functions as a GTP-dependent effector protein in thrombin-induced platelet cytoskeletal reorganization. Blood. 2003;101(8):3021–8. Epub 2003/01/08. doi: 10.1182/blood-2002-09-2807 2002-09-2807 [pii]. . 12515716 10.1182/blood-2002-09-2807

[12] Qualls JE, Kaplan AM, van Rooijen N, Cohen DA. Suppression of experimental colitis by intestinal mononuclear phagocytes. Journal of leukocyte biology. 2006;80(4):802–15. 10.1189/jlb.1205734 . 16888083

[13] Heid CA, Stevens J, Livak KJ, Williams PM. Real time quantitative PCR. Genome Res. 1996;6(10):986–94. . 8908518 10.1101/gr.6.10.986

[14] Livak KJ, Schmittgen TD. Analysis of relative gene expression data using real-time quantitative PCR and the 2(-Delta Delta C(T)) Method. Methods. 2001;25(4):402–8. 10.1006/meth.2001.1262 . 11846609

[15] Joseph F. LaComb JML, Valentina A. Schmidt. IQGAP2 inhibits cell proliferation and migration in hepatocellular carcinoma [abstract]. In: Proceedings of the 103rd Annual Meeting of the American Association for Cancer Research; 2012 March 31-April 4; Chicago, IL, Abstract nr 3989 2012.

[16] Bouma G, Strober W. The immunological and genetic basis of inflammatory bowel disease. Nature reviews Immunology. 2003;3(7):521–33. 10.1038/nri1132 . 12876555

[17] Mahler M, Bristol IJ, Leiter EH, Workman AE, Birkenmeier EH, Elson CO, et al Differential susceptibility of inbred mouse strains to dextran sulfate sodium-induced colitis. Am J Physiol. 1998;274(3 Pt 1):G544-51. . 9530156 10.1152/ajpgi.1998.274.3.G544

[18] Schreiber S, Nikolaus S, Hampe J. Activation of nuclear factor kappa B inflammatory bowel disease. Gut. 1998;42(4):477–84. 9616307 10.1136/gut.42.4.477 PMC1727068

[19] Marrero JA, Matkowskyj KA, Yung K, Hecht G, Benya RV. Dextran sulfate sodium-induced murine colitis activates NF-kappaB and increases galanin-1 receptor expression. Am J Physiol Gastrointest Liver Physiol. 2000;278(5):G797–804. . 10801272 10.1152/ajpgi.2000.278.5.G797

[20] Bouwmeester T, Bauch A, Ruffner H, Angrand PO, Bergamini G, Croughton K, et al A physical and functional map of the human TNF-alpha/NF-kappa B signal transduction pathway. Nat Cell Biol. 2004;6(2):97–105. 10.1038/ncb1086 . 14743216

[21] Carmody RJ, Chen YH. Nuclear factor-kappaB: activation and regulation during toll-like receptor signaling. Cellular & molecular immunology. 2007;4(1):31–41. . 17349209

[22] Medzhitov R, Preston-Hurlburt P, Janeway CA Jr. A human homologue of the Drosophila Toll protein signals activation of adaptive immunity. Nature. 1997;388(6640):394–7. 10.1038/41131 . 9237759

[23] Cario E. Toll-like receptors in inflammatory bowel diseases: a decade later. Inflammatory bowel diseases. 2010;16(9):1583–97. 10.1002/ibd.21282 20803699 PMC2958454

[24] Ortega-Cava CF, Ishihara S, Rumi MA, Kawashima K, Ishimura N, Kazumori H, et al Strategic compartmentalization of Toll-like receptor 4 in the mouse gut. J Immunol. 2003;170(8):3977–85. . 12682225 10.4049/jimmunol.170.8.3977

[25] Lu YC, Yeh WC, Ohashi PS. LPS/TLR4 signal transduction pathway. Cytokine. 2008;42(2):145–51. 10.1016/j.cyto.2008.01.006 . 18304834

[26] Libermann TA, Baltimore D. Activation of interleukin-6 gene expression through the NF-kappa B transcription factor. Mol Cell Biol. 1990;10(5):2327–34. 2183031 10.1128/mcb.10.5.2327-2334.1990 PMC360580

[27] Xavier RJ, Podolsky DK. Unravelling the pathogenesis of inflammatory bowel disease. Nature. 2007;448(7152):427–34. 10.1038/nature06005 . 17653185

[28] Yan Y, Kolachala V, Dalmasso G, Nguyen H, Laroui H, Sitaraman SV, et al Temporal and spatial analysis of clinical and molecular parameters in dextran sodium sulfate induced colitis. PLoS One. 2009;4(6):e6073 10.1371/journal.pone.0006073 19562033 PMC2698136

[29] Raddatz D, Bockemuhl M, Ramadori G. Quantitative measurement of cytokine mRNA in inflammatory bowel disease: relation to clinical and endoscopic activity and outcome. European journal of gastroenterology & hepatology. 2005;17(5):547–57. 10.1007/s00535-009-0024-z 15827446

[30] Grivennikov S, Karin E, Terzic J, Mucida D, Yu GY, Vallabhapurapu S, et al IL-6 and Stat3 are required for survival of intestinal epithelial cells and development of colitis-associated cancer. Cancer Cell. 2009;15(2):103–13. 10.1016/j.ccr.2009.01.001 19185845 PMC2667107

[31] Bollrath J, Phesse TJ, von Burstin VA, Putoczki T, Bennecke M, Bateman T, et al gp130-mediated Stat3 activation in enterocytes regulates cell survival and cell-cycle progression during colitis-associated tumorigenesis. Cancer Cell. 2009;15(2):91–102. 10.1016/j.ccr.2009.01.002 . 19185844

[32] Buettner R, Mora LB, Jove R. Activated STAT signaling in human tumors provides novel molecular targets for therapeutic intervention. Clin Cancer Res. 2002;8(4):945–54. . 11948098

[33] Dieleman LA, Ridwan BU, Tennyson GS, Beagley KW, Bucy RP, Elson CO. Dextran sulfate sodium-induced colitis occurs in severe combined immunodeficient mice. Gastroenterology. 1994;107(6):1643–52. . 7958674 10.1016/0016-5085(94)90803-6

[34] Schmidt VA. Watch the GAP: Emerging Roles for IQ Motif-Containing GTPase-Activating Proteins IQGAPs in Hepatocellular Carcinoma. International journal of hepatology. 2012;2012:958673 Epub 2012/09/14. 10.1155/2012/958673 22973521 PMC3438877

[35] Arbibe L, Mira JP, Teusch N, Kline L, Guha M, Mackman N, et al Toll-like receptor 2-mediated NF-kappa B activation requires a Rac1-dependent pathway. Nature immunology. 2000;1(6):533–40. 10.1038/82797 . 11101877

[36] Uno JK, Rao KN, Matsuoka K, Sheikh SZ, Kobayashi T, Li F, et al Altered macrophage function contributes to colitis in mice defective in the phosphoinositide-3 kinase subunit p110delta. Gastroenterology. 2010;139(5):1642–53, 53 e1-6. 10.1053/j.gastro.2010.07.008 20637203 PMC2967619

[37] Perona R, Montaner S, Saniger L, Sanchez-Perez I, Bravo R, Lacal JC. Activation of the nuclear factor-kappaB by Rho, CDC42, and Rac-1 proteins. Genes Dev. 1997;11(4):463–75. . 9042860 10.1101/gad.11.4.463

[38] Muise AM, Walters T, Xu W, Shen-Tu G, Guo CH, Fattouh R, et al Single nucleotide polymorphisms that increase expression of the guanosine triphosphatase RAC1 are associated with ulcerative colitis. Gastroenterology. 2011;141(2):633–41. 10.1053/j.gastro.2011.04.057 21684284 PMC3152589

[39] Osman MA, Sarkar FH, Rodriguez-Boulan E. A molecular rheostat at the interface of cancer and diabetes. Biochim Biophys Acta. 2013;1836(1):166–76. 10.1016/j.bbcan.2013.04.005 23639840 PMC3667713

[40] Sbroggio M, Carnevale D, Bertero A, Cifelli G, De Blasio E, Mascio G, et al IQGAP1 regulates ERK1/2 and AKT signaling in the heart and sustains functional remodeling upon pressure overload. Cardiovasc Res. 2011;91(3):456–64. Epub 2011/04/16. doi: cvr103 [pii] 10.1093/cvr/cvr103 . 21493702 PMC3294280

[41] Cai J, Zhang N, Zheng Y, de Wilde RF, Maitra A, Pan D. The Hippo signaling pathway restricts the oncogenic potential of an intestinal regeneration program. Genes Dev. 2010;24(21):2383–8. 10.1101/gad.1978810 21041407 PMC2964748

[42] Barry ER, Morikawa T, Butler BL, Shrestha K, de la Rosa R, Yan KS, et al Restriction of intestinal stem cell expansion and the regenerative response by YAP. Nature. 2013;493(7430):106–10. 10.1038/nature11693 23178811 PMC3536889

[43] Anakk S, Bhosale M, Schmidt VA, Johnson RL, Finegold MJ, Moore DD. Bile Acids Activate YAP to Promote Liver Carcinogenesis. Cell reports. 2013;5(4):1060–9. Epub 2013/11/26. 10.1016/j.celrep.2013.10.030 . 24268772 PMC3961013

[44] Abreu MT. Toll-like receptor signalling in the intestinal epithelium: how bacterial recognition shapes intestinal function. Nature reviews Immunology. 2010;10(2):131–44. 10.1038/nri2707 . 20098461

[45] Okabe Y, Medzhitov R. Tissue-specific signals control reversible program of localization and functional polarization of macrophages. Cell. 2014;157(4):832–44. 10.1016/j.cell.2014.04.016 24792964 PMC4137874

[46] Sanjuan MA, Dillon CP, Tait SW, Moshiach S, Dorsey F, Connell S, et al Toll-like receptor signalling in macrophages links the autophagy pathway to phagocytosis. Nature. 2007;450(7173):1253–7. 10.1038/nature06421 . 18097414

[47] Turgeon N, Gagne JM, Blais M, Gendron FP, Boudreau F, Asselin C. The acetylome regulators Hdac1 and Hdac2 differently modulate intestinal epithelial cell dependent homeostatic responses in experimental colitis. American journal of physiology Gastrointestinal and liver physiology. 2014;306(7):G594–605. Epub 2014/02/15. 10.1152/ajpgi.00393.2013 . 24525021

[48] Wu F DF, Arendovich N, Zhan J, Huang Y, Kwon JH. GSE59648. Microarray submission date Jul 22, 2014.

[49] Salcedo R, Worschech A, Cardone M, Jones Y, Gyulai Z, Dai RM, et al MyD88-mediated signaling prevents development of adenocarcinomas of the colon: role of interleukin 18. J Exp Med. 2010;207(8):1625–36. 10.1084/jem.20100199 20624890 PMC2916129

[50] Jameson KL, Mazur PK, Zehnder AM, Zhang J, Zarnegar B, Sage J, et al IQGAP1 scaffold-kinase interaction blockade selectively targets RAS-MAP kinase-driven tumors. Nat Med. 2013;19(5):626–30. Epub 2013/04/23. 10.1038/nm.3165 . 23603816 PMC4190012

[51] McLean DT, Lundy FT, Timson DJ. IQ-motif peptides as novel anti-microbial agents. Biochimie. 2013;95(4):875–80. 10.1016/j.biochi.2012.12.004 . 23238369

