# Supplementary figures and images for: IQ Motif-Containing GTPase-Activating Protein 2 (IQGAP2) Is a Novel Regulator of Colonic Inflammation in Mice
Source: PLoS One. 2015 Jun 5;10(6):e0129314. doi: 10.1371/journal.pone.0129314 (PMC4457730; doi:10.1371/journal.pone.0129314)

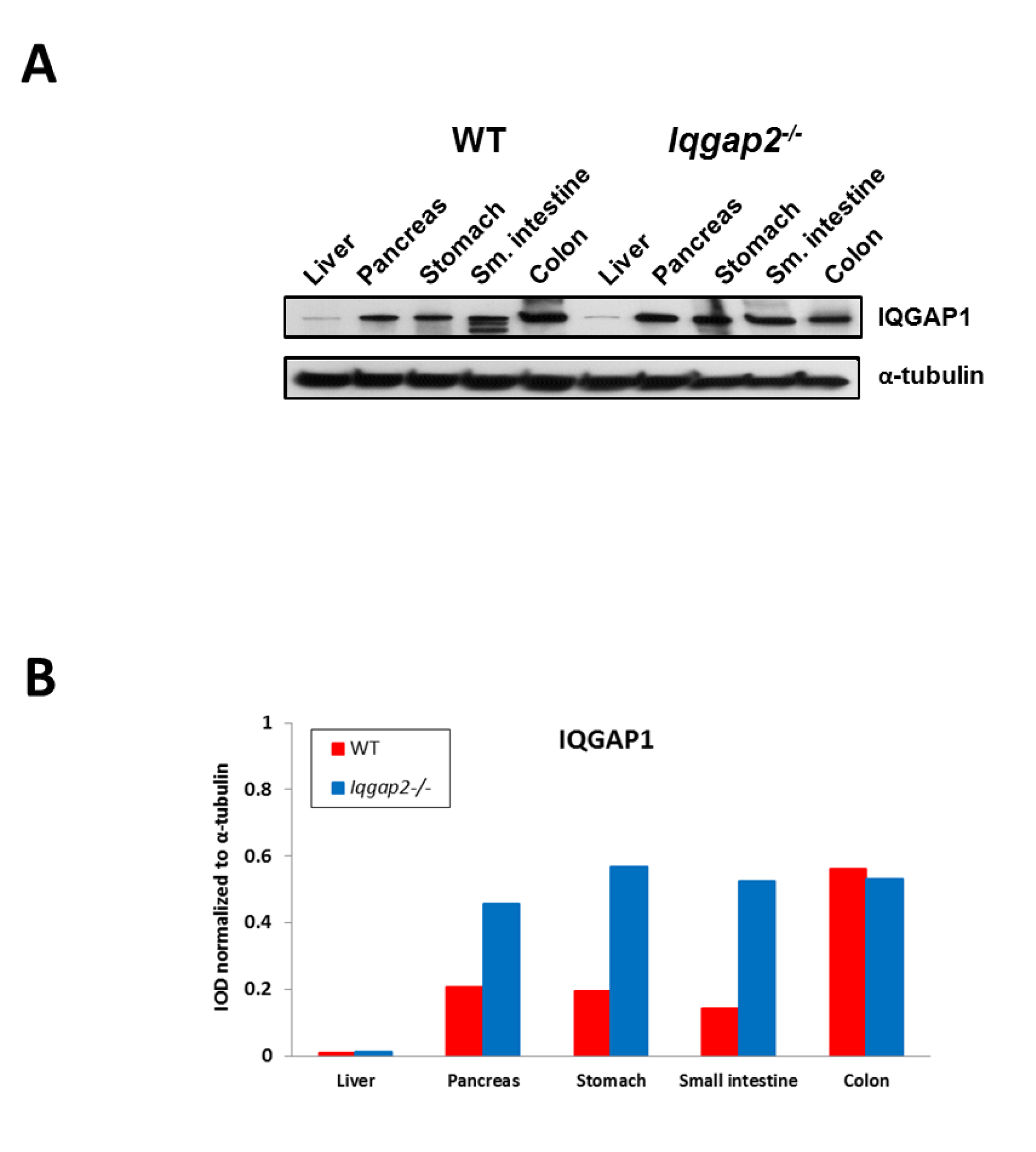

Supplement: S1 Fig — A. Immunoblot showing IQGAP1 expression in organs of mouse digestive tract. B. Densitometric quantification of IQGAP1 protein levels in WT vs. Iqgap2 -/- organs normalized to α-tubulin. (TIF) [file pone.0129314.s001.tif]

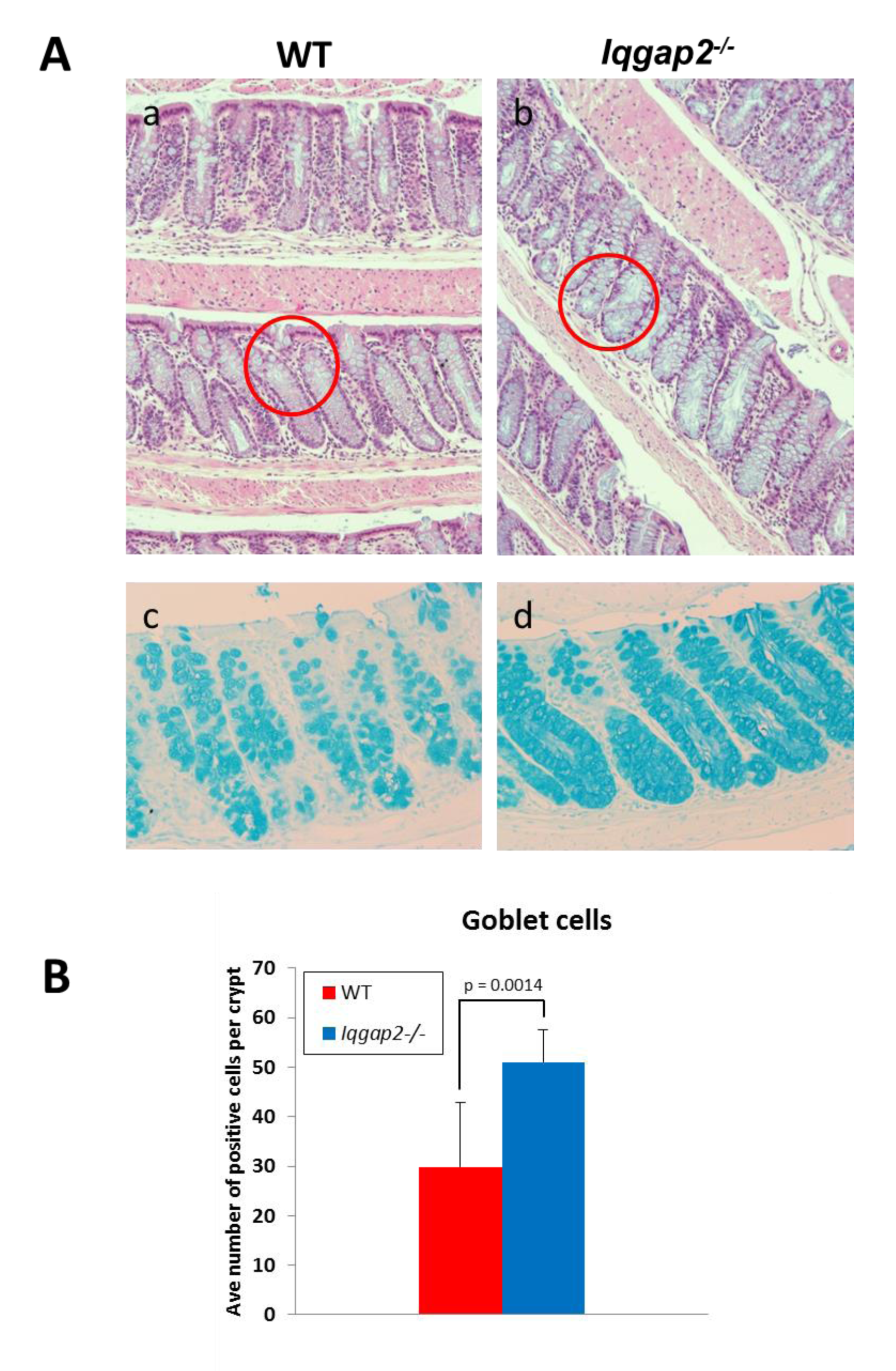

Supplement: S2 Fig — A. H&E (panels a, b) and Alcian Blue (panels c, d) stainings. Representative images of N = 3 per genotype are shown. Characteristic crypts with distinct size of goblet cells are circled. B. Numbers of goblet cells were counted per crypt in six random fields and data presented as mean ± SD. A p-value indicating significant difference is shown. (TIF) [file pone.0129314.s002.tif]

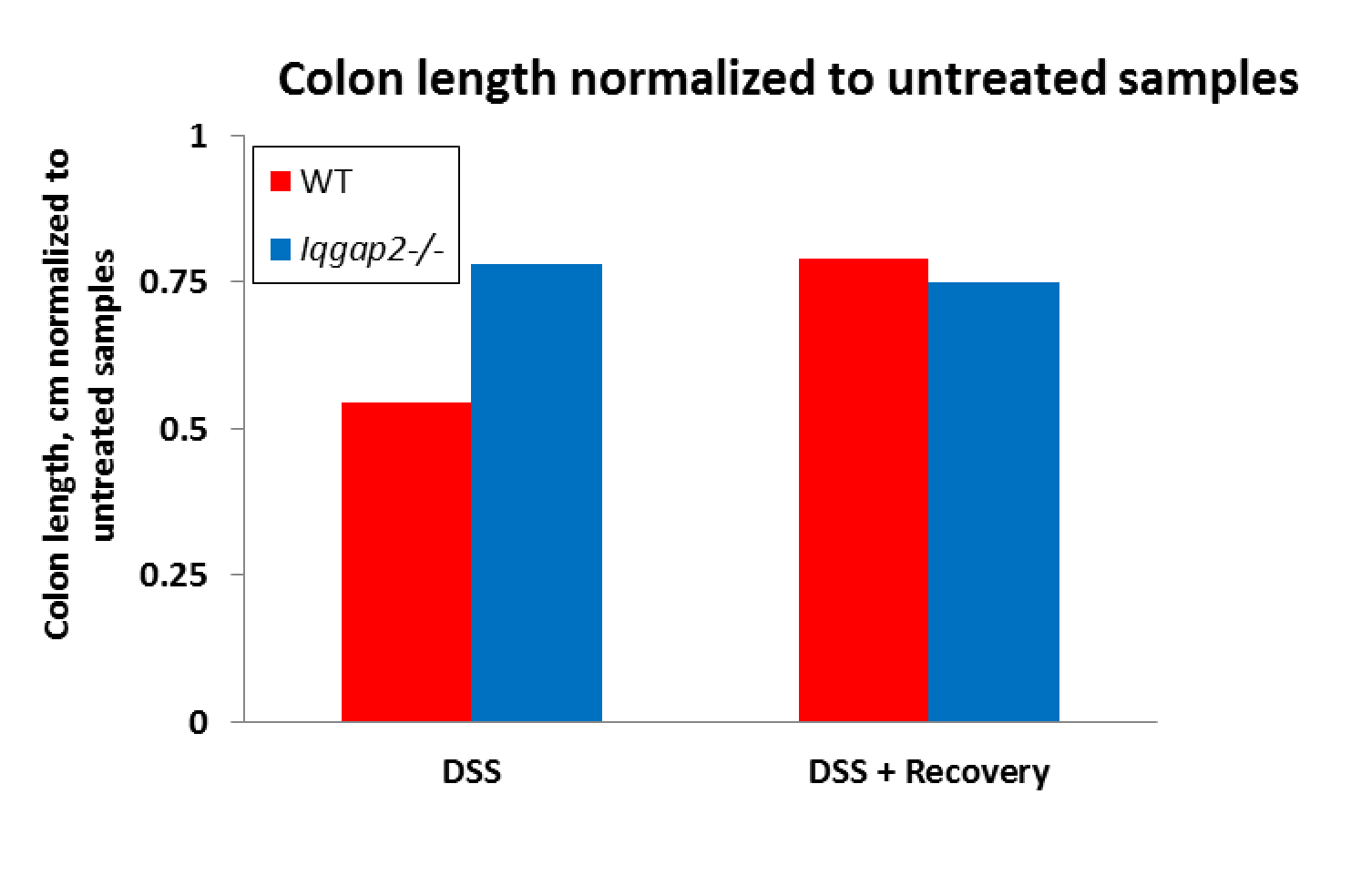

Supplement: S3 Fig — Colon length of WT and Iqgap2 -/- mice from two treatment groups, DSS-treated and DSS+Recovery, was normalized to colon length of untreated mice of respective genotypes. (TIF) [file pone.0129314.s003.tif]

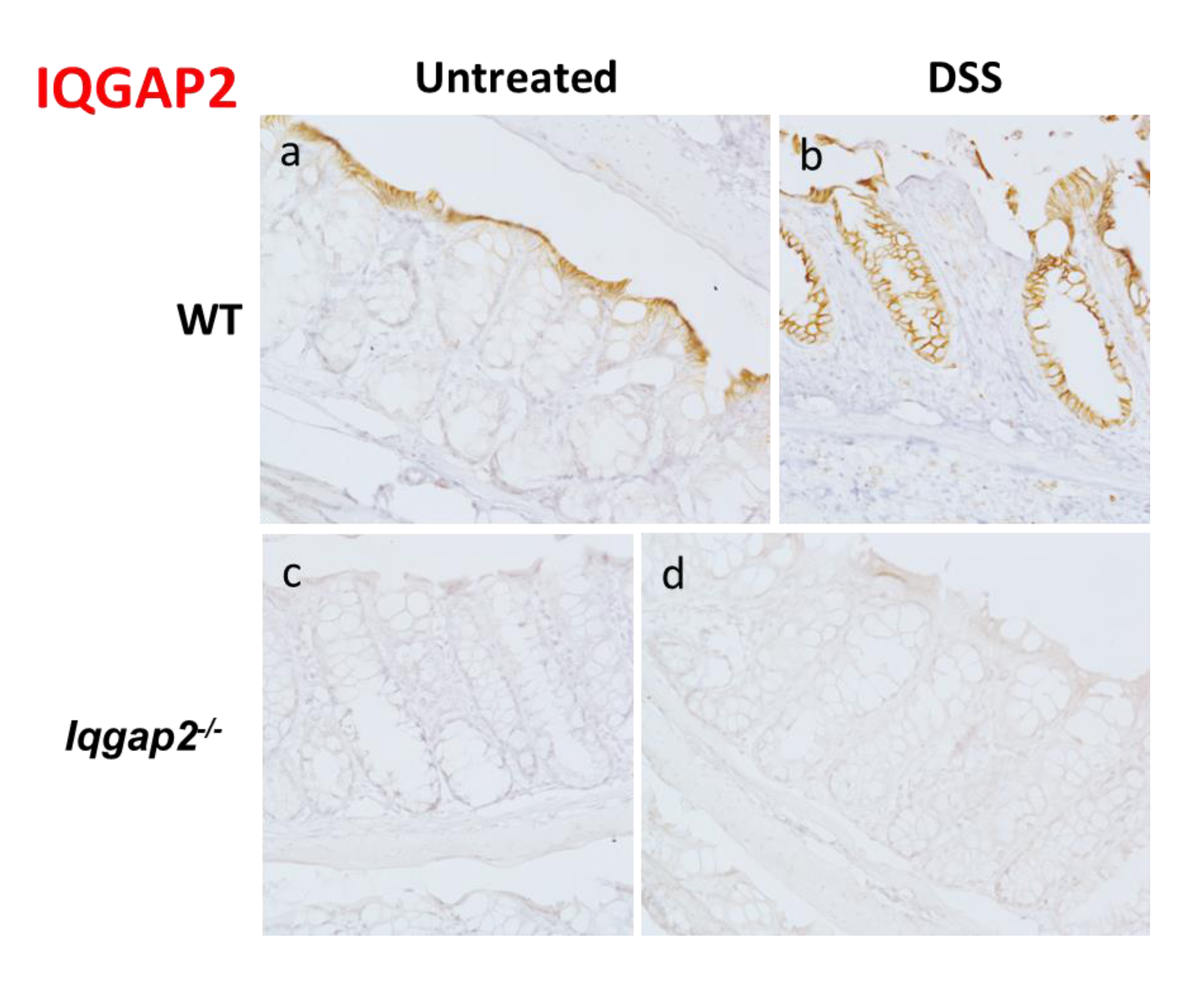

Supplement: S4 Fig — Representative images of N = 5 are shown. Magnification is 200 X. (TIF) [file pone.0129314.s004.tif]

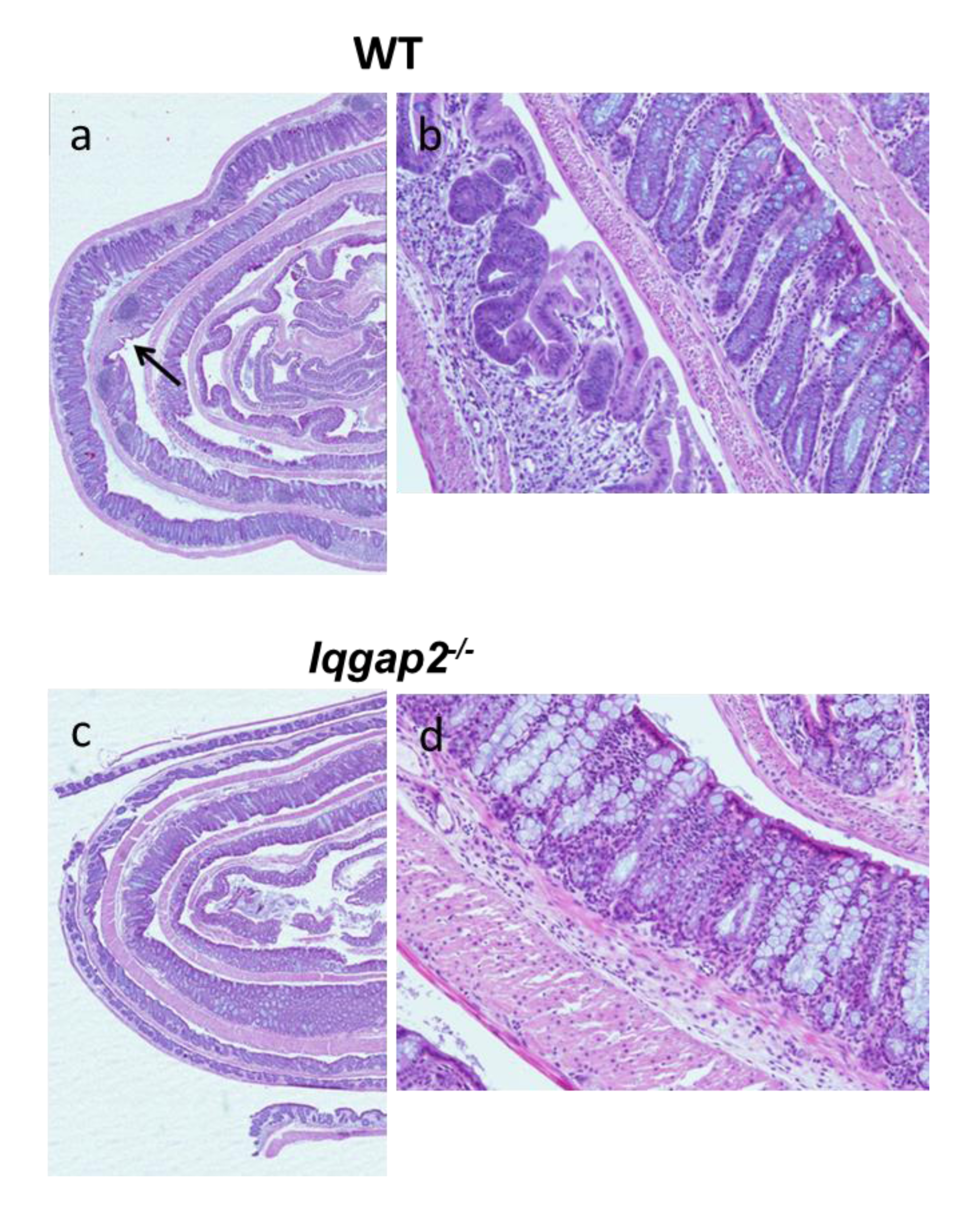

Supplement: S5 Fig — N = 5 per genotype; experiment was repeated three times; the results of one representative experiment are shown. Panels b and d show higher magnification of samples in panels a and c. An unrepaired area of epithelium loss is marked with a black arrow. (TIF) [file pone.0129314.s005.tif]

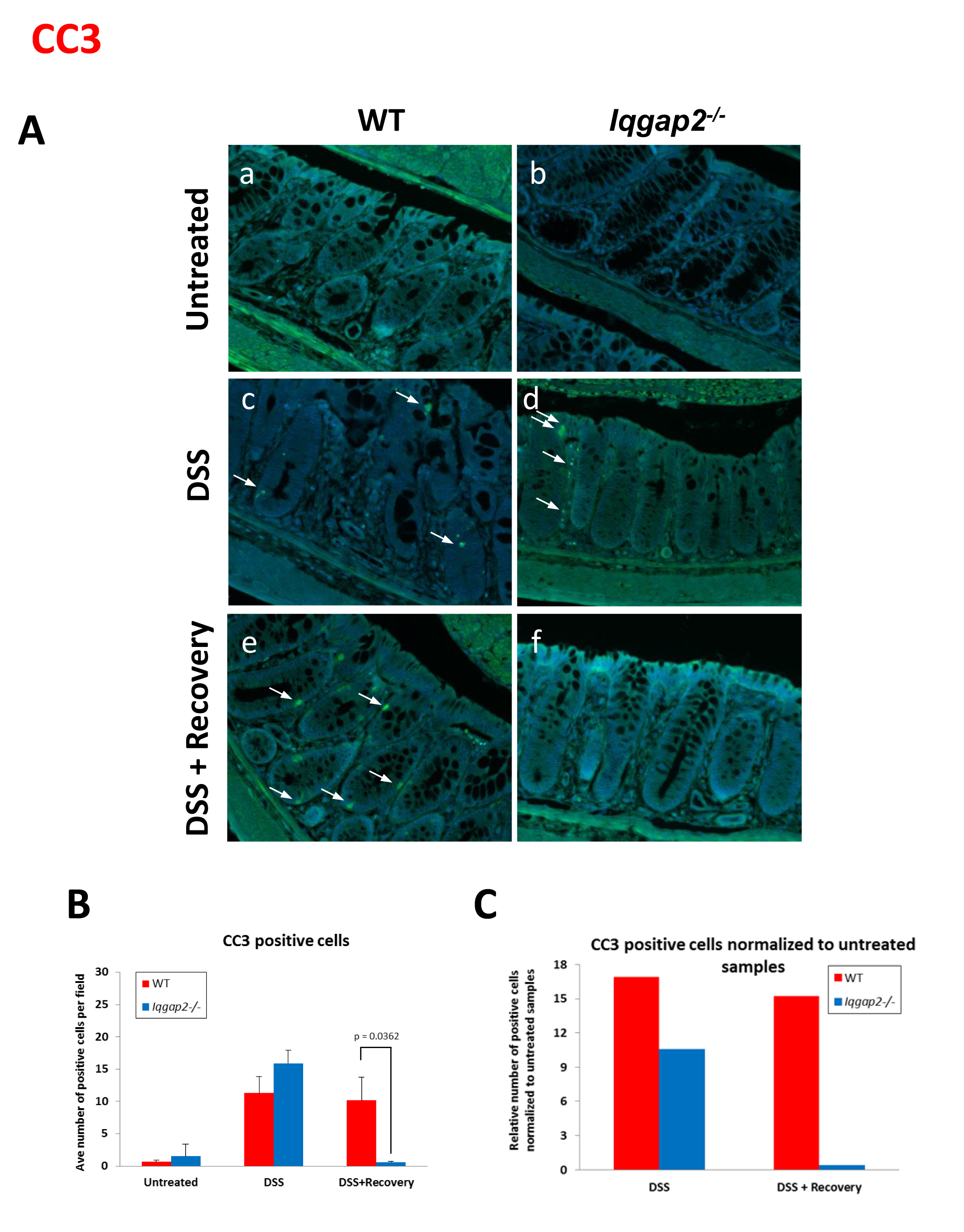

Supplement: S6 Fig — A. DSS induces apoptosis at a similar rate in Iqgap2 -/- colons compared to WT controls as evident by IF using cleaved caspase-3 (CC3) antibody. Representative images of colon sections from untreated and DSS-treated WT (panels a, c) and Iqgap2 -/- (panels b, d) mice are shown. Representative images of colons from the DSS+Recovery group (panels e, f) are also shown. Images are overlays of CC3 IF (green) and DAPI (blue) staining. Images are representative of N = 3 per group. White arrows indicate characteristic CC3-positive cells. B. Quantification of CC3-positive cells in WT and Iqgap2 -/- colons from mice from the three treatment groups: untreated, DSS-treated and DSS and Recovery. CC3-positive cells were counted in crypts in six random fields. Data are presented as mean ± SD per crypt. A p-value indicating statistically significant difference is shown. C. Numbers of CC3-positive cells in colons from the DSS and DSS+Recovery groups normalized to numbers of CC3-positive cells in untreated colons. (TIF) [file pone.0129314.s006.tif]

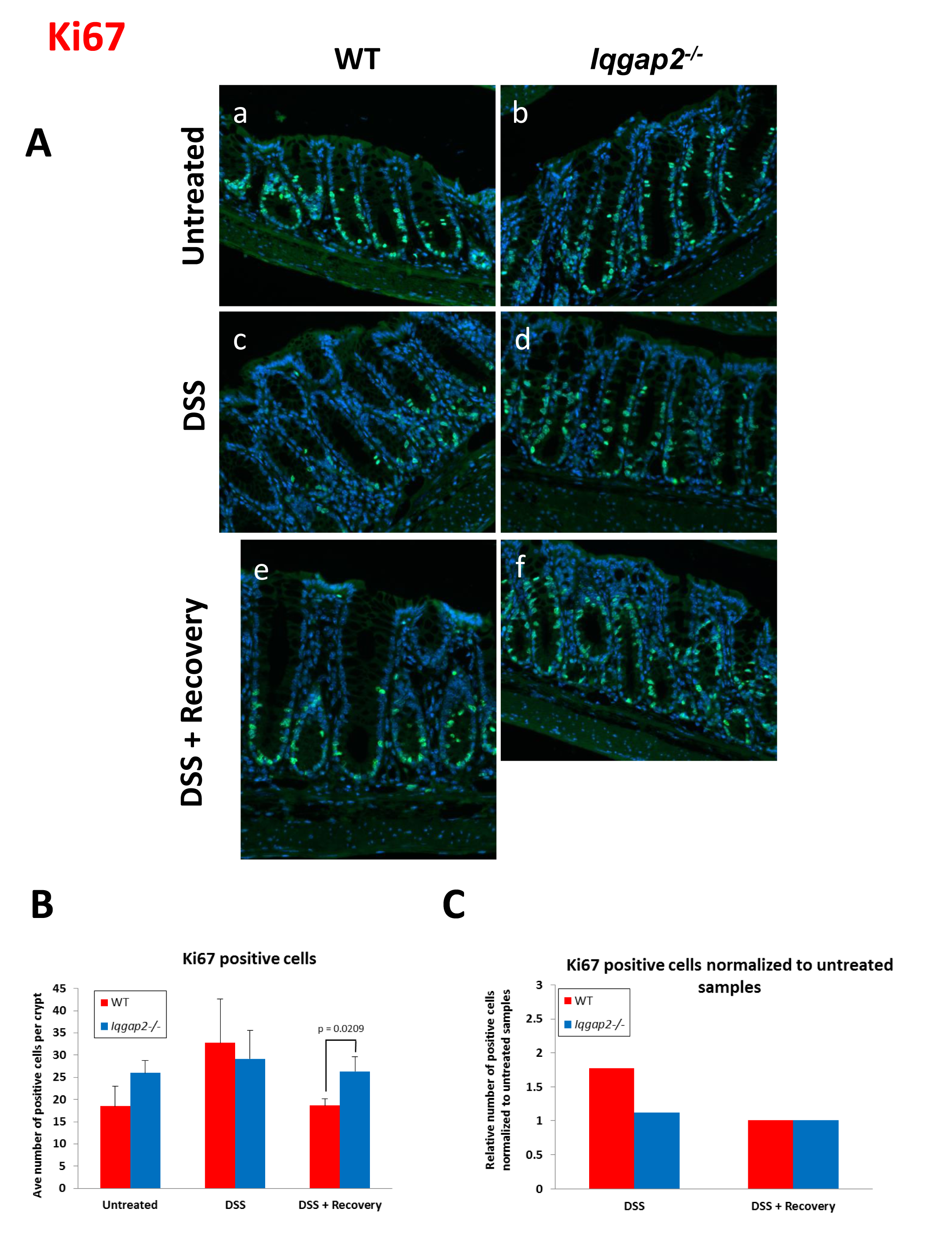

Supplement: S7 Fig — A. Ki67 IF of colons from the untreated, DSS-treated and DSS+Recovery mouse groups. Images are overlays of Ki67 IF (green) and DAPI (blue) staining. Images are representative of N = 3 per group. B. Quantification of Ki67-positive cells was conducted as described for CC3 in S6 Fig. Magnification is 200 X. C. Numbers of Ki67-positive cells in colons from the DSS-treated and DSS+Recovery groups normalized to numbers of Ki67-positive cells in untreated colons. (TIF) [file pone.0129314.s007.tif]

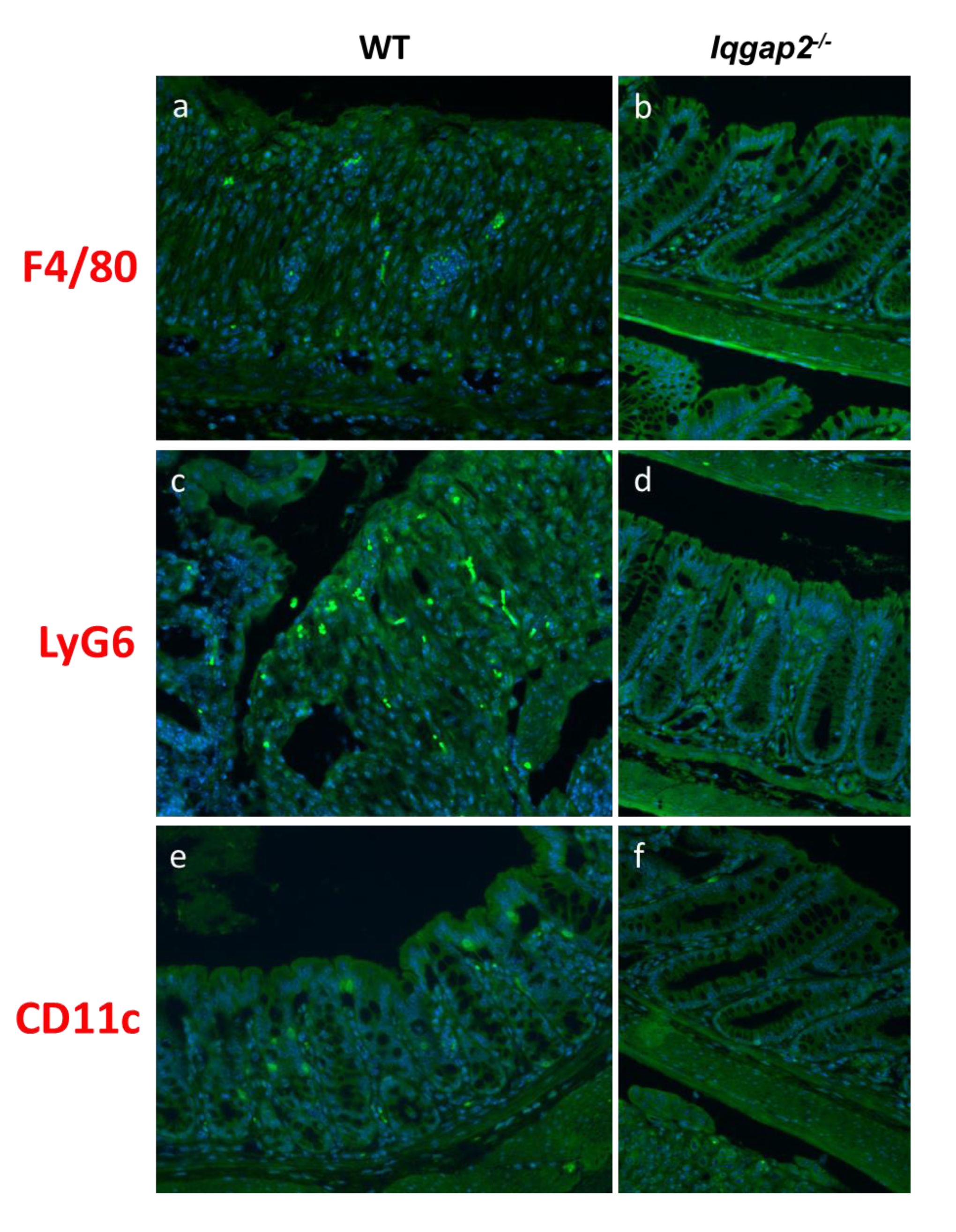

Supplement: S8 Fig — Representative images of IF for F4/80 (panels a, b), Ly-6G (panels c, d) and CD11c (panels e, f) using colon sections from DSS-treated WT and Iqgap2 -/- mice, N = 3 per group. Images are overlays of IF with antibodies of interest (green) and DAPI (blue) staining. Magnification is 200 X. (TIF) [file pone.0129314.s008.tif]

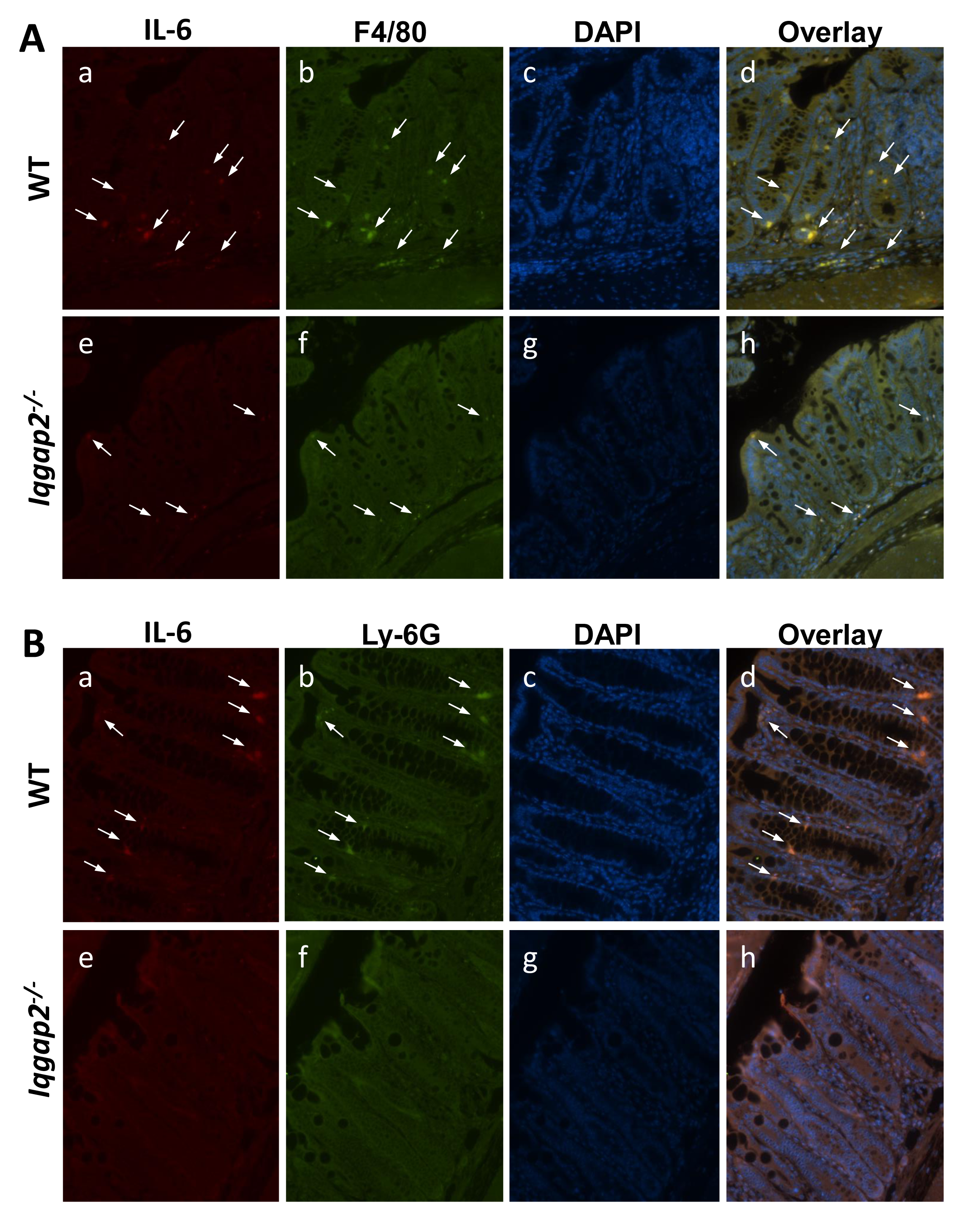

Supplement: S9 Fig — Representative images of IF for IL-6 (red, panels a, e), either F4/80 or Ly-6G (green, panels b, f), and DAPI (blue, panels c, g) using colon sections from DSS-treated WT and Iqgap2 -/- mice are shown, N = 3 per group. Images in panels d, h are overlays of three stainings: IL-6, either F4/80 or Ly-6G, and DAPI. White arrows indicate characteristic positive cells. Magnification is 200 X. (TIF) [file pone.0129314.s009.tif]

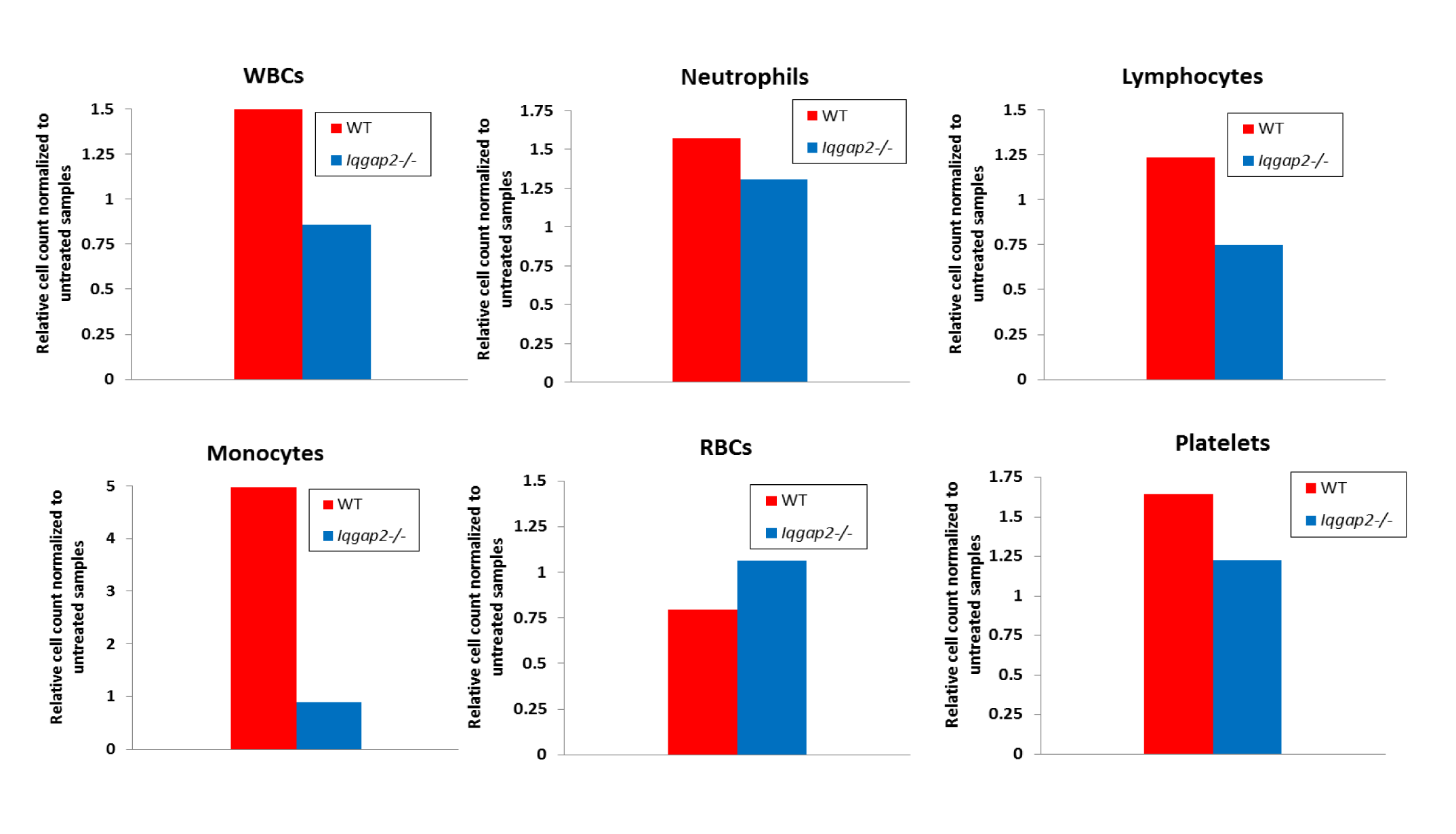

Supplement: S10 Fig — CBCs of WT and Iqgap2 -/- mice from the DSS-treated group were normalized to CBCs of untreated mice of respective genotypes. (TIF) [file pone.0129314.s010.tif]

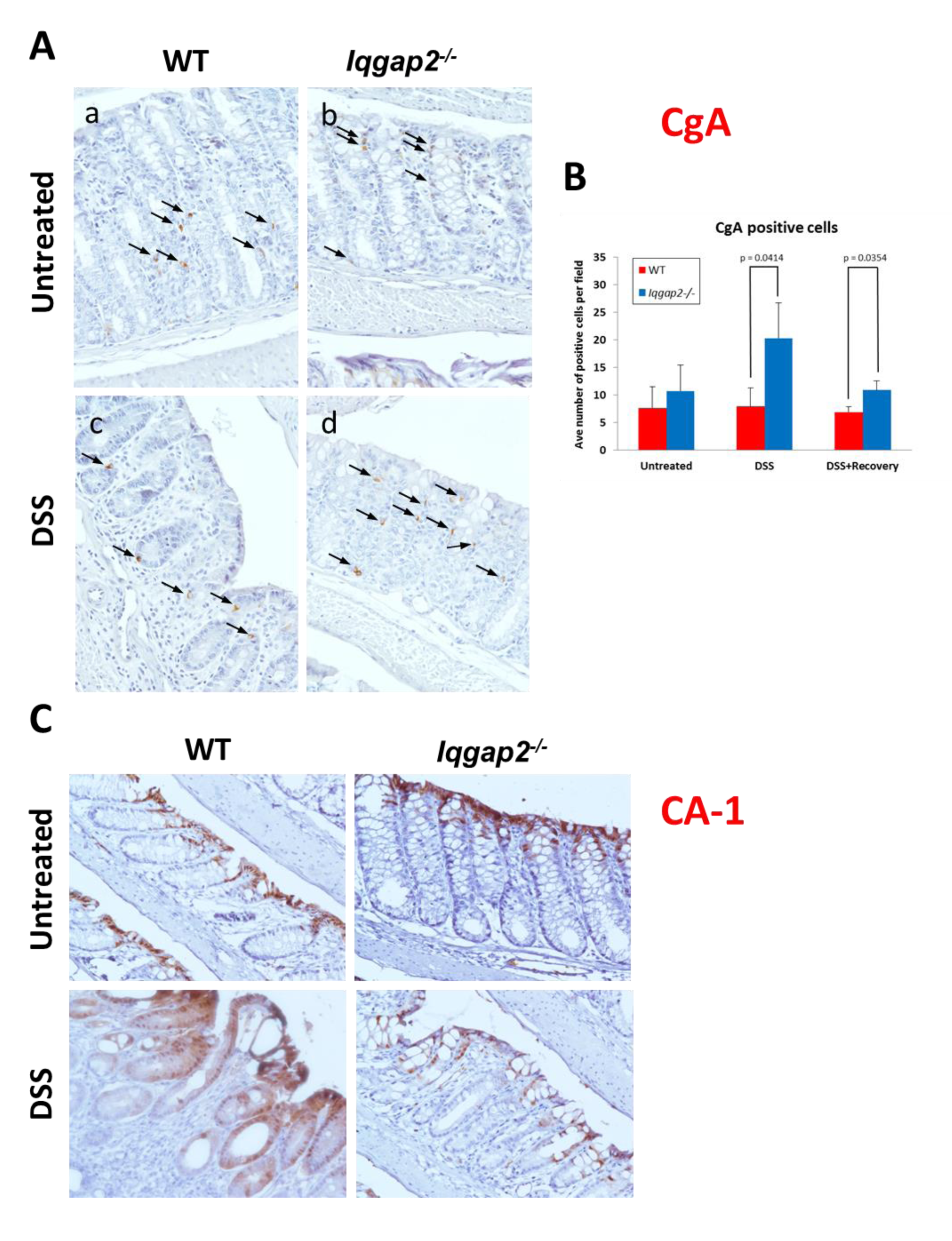

Supplement: S11 Fig — A. Representative images of WT and Iqgap2 -/- colons untreated and after DSS treatment, N = 5 per group per genotype. Magnification is 200 X. Characteristic positive cells are pointed with black arrows. B. Quantification of CgA-positive cells. Positive cells were counted in six random fields. Data are presented as mean ± SD. P-values indicating statistically significant difference are shown. C. IHC of carbonic anhydrase-1 (CA-1) in the same samples as in A. (TIF) [file pone.0129314.s011.tif]
