# Supplementary material for: IQ Motif-Containing GTPase-Activating Protein 2 (IQGAP2) Is a Novel Regulator of Colonic Inflammation in Mice
Source: PLoS One. 2015 Jun 5;10(6):e0129314. doi: 10.1371/journal.pone.0129314 (PMC4457730; doi:10.1371/journal.pone.0129314)
Supplement: S1 Table — Meta-analysis was conducted to assess changes in Iqgap2 mRNA levels in human IBD, CRC, and mouse DSS- and AOM-DSS-induced colitis models. (DOCX) [file pone.0129314.s012.docx]

| Human IBD | Fold change | p-value |
| --- | --- | --- |
| **Genome-wide gene expression differences between Crohn’s and ulcerative colitis from endoscopic pinch biopsies, GSE6731 [**[**50**](#_ENREF_50)**]:**  Colon from UC patients, affected tissue vs healthy controls  Colon from CD patients, affected tissue vs healthy controls  Colon from UC patients, affected tissue vs unaffected tissue  Colon from CD patients, affected tissue vs unaffected tissue | -1.8  -1.48  -1.82  -1.42 | 0.0234  0.0229  0.0351  0.0184 |
| **Expression profiling in inflammatory bowel disease, GSE1710 [**[**51**](#_ENREF_51)**]:**  Sigmoid colon from CD patients (N=10) vs normal (N=10)  Sigmoid colon from UC patients (N=11) vs normal (N=10) | -1.21  -1.21 | 0.0179  0.0186 |
| **Colon epithelial biopsies of ulcerative colitis patients, GDS3268 [**[**52**](#_ENREF_52)**]:** Inflamed colon from non-IBD patients (N=8) vs healthy controls (N=23)  Severely inflamed colon from UC patients (N=67) vs healthy controls (N=8) | -1.3  -1.4 | 0.0006  0.0006 |
| **Inflammation, adenoma and cancer: objective classification of colon biopsy specimens with gene expression signature, GSE4183 [**[**53**](#_ENREF_53)**]:** Colon biopsies from IBD patients (N=15) vs healthy colon (N=8) | -1.49 | 0.0006 |
| **Definition of an ulcerative colitis pre-inflammatory state, GSE9452 [**[**54**](#_ENREF_54)**]:**  UC colonic mucosa with inflammation vs healthy control  UC colonic mucosa with inflammation vs non-inflamed UC | -1.78  -1.55 | 0.0011  0.0039 |
| **Expression data from intestinal mucosa of patients with UC, GSE38713 [**[**55**](#_ENREF_55)**]:**  Colonic mucosa from patients with active UC vs healthy controls  Colonic mucosa from patients with inactive UC vs healthy controls | -1.72  -1.55 | 6.7x10^-5^  1.5x10^-5^ |
| **Genome-wide analysis of Crohn's disease and ulcerative colitis biopsy samples, GSE36807 [**[**56**](#_ENREF_56)**]:**  Intestinal biopsy from UC patients vs healthy controls  Intestinal biopsy from CD patients vs healthy controls – **not significant** | -1.39 | 0.0196 |
| **Human colon expression in healthy controls and UC, GSE10191 [**[**57**](#_ENREF_57)**]:**  Colon from UC patients vs healthy controls | -1.78 | 0.0017 |
| Human CRC | Fold change | p-value |
| **Inflammation, adenoma and cancer: objective classification of colon biopsy specimens with gene expression signature, GSE4183 [**[**53**](#_ENREF_53)**]:**  Colon biopsies from patients with colon adenoma (N=15) vs healthy controls (N=8)  Colon biopsies from patients with colorectal carcinoma (N=15) vs healthy controls (N=8) | -1.79  -1.51 | 0.0001  0.0059 |
| **Colorectal cancer profiling and outcome prediction after resection and adjuvant chemotherapy, GSE17538 [**[**58**](#_ENREF_58)**]:**  Stage III CRC tumors from patients who died of cancer in 1 year vs stage III CRC tumors from patients who died after 3 years (N=244) | -2.7 | 0.001 |
| **Whole genome analysis for liver metastasis gene signatures in colorectal** **cancer, GSE6988 [**[**59**](#_ENREF_59)**]:**  CRC liver metastasis adenocarcinoma vs CRC primary adenocarcinoma (N=123) | +2.64 | 0.0036 |
| **Analysis of colorectal tissue from APC- and MYH-associated polyposis patients, GSE9689 [**[**60**](#_ENREF_60)**]:**  Adenomatous polyposis biopsies with monoallelic APC mutation vs adjacent normal tissue (N=78) | -1.58 | 0.005 |
| Mouse DSS- and AOM-DSS-induced colitis | Fold change | p-value |
| **Colon epithelia from HDAC2 conditional knockout mice, GSE54785 [**[**61**](#_ENREF_61)**]:**  Colon epithelia from HDAC2 conditional KO mice vs WT (N=6) | +1.21 | 0.0021 |
| **Divergent influence of microRNA-21 deletion on murine colitis phenotypes, GSE59648 [**[**62**](#_ENREF_62)**]:**  Colon mucosae from miR-21 KO mice vs WT | +1.77 | 0.0168 |
| **MyD88-mediated signaling prevents development of adenocarcinomas of the colon via interleukin-18, GSE19739 [**[**63**](#_ENREF_63)**]:**  Colon of 6-8 weeks MyD88-KO mice with AOM/DSS-induced colitis vs healthy colon of MyD88-KO mice (N=32)  Colon of 6-8 weeks MyD88-KO mice with AOM/DSS-induced colitis vs colon of wild type mice AOM/DSS-induced colitis (N=32) | +1.75  +1.56 | 0.0001  0.0025 |
